# Mastering the Wrinkling of Self-supported Graphene

**DOI:** 10.1038/s41598-017-10153-z

**Published:** 2017-08-30

**Authors:** Barbara Pacakova, Tim Verhagen, Milan Bousa, Uwe Hübner, Jana Vejpravova, Martin Kalbac, Otakar Frank

**Affiliations:** 1 Institute of Physics of the CAS, v.v.i, Na Slovance 2, CZ-182 21 Prague 8, Czech Republic; 2 J. Heyrovsky Institute of Physical Chemistry of the CAS, v.v.i., Dolejskova 3, CZ-182 23 Prague 8, Czech Republic; 3 Department of Inorganic Chemistry, Faculty of Science, Charles University, Albertov 6, CZ-128 43 Prague 2, Czech Republic; 4 Leibniz Institute of Photonic Technology (IPHT), PO. Box 100239, D-07702 Jena, Germany; 5 Department of Condensed Matter Physics, Faculty of Mathematics and Physics, Charles University, V Holesovickach 2, CZ-180 00 Prague 8, Czech Republic

## Abstract

We present an approach that allows for the preparation of well-defined large arrays of graphene wrinkles with predictable geometry. Chemical vapor deposition grown graphene transferred onto hexagonal pillar arrays of SiO_2_ with sufficiently small interpillar distance forms a complex network of two main types of wrinkle arrangements. The first type is composed of arrays of aligned equidistantly separated parallel wrinkles propagating over large distances, and originates from line interfaces in the graphene, such as thin, long wrinkles and graphene grain boundaries. The second type of wrinkle arrangement is composed of non-aligned short wrinkles, formed in areas without line interfaces. Besides the presented hybrid graphene topography with distinct wrinkle geometries induced by the pre-patterned substrate, the graphene layers are suspended and self-supporting, exhibiting large surface area and negligible doping effects from the substrate. All these properties make this wrinkled graphene a promising candidate for a material with enhanced chemical reactivity useful in nanoelectronic applications.

## Introduction

In recent years, graphene-based research has moved from investigating flat ideal two dimensional (2D) graphene to exploring structures that are topologically distorted^1–7^. Corrugating graphene via creating folds, crumples, ripples, and wrinkles produces a material that combines the intrinsic (the highest Young’s modulus observed, the highest room temperature carrier mobility) and enhanced properties of graphene.

Because wavy-like structures are observed everywhere in nature^8^, corrugated graphene exists even if it is not exposed to artificial manipulation with the topography. In fact, real graphene is never completely flat; it always exhibits surface corrugations, which prevents the monolayer from collapsing^1, 2^. In general, surface corrugations manifesting as a local topological disorder significantly modify the electrical, optical, and chemical properties of graphene in comparison to the intrinsic properties of non-corrugated graphene^3, 9, 10^ by varying the electrostatic potential of the layer and by further modifying the band structure. For example, a change in the relative position of the Dirac point (described by energy *E*_D_) with respect to the Fermi level (with energy *E*_F_)^11^, the creation of the mid-gap states^12^, and trigonal warping^13^ have been observed. Moreover, the creation of surface corrugations in graphene results in a change in the charge transport^5^, the suppression of weak localization^11^, the appearance of electron–hole puddles^14, 15^, band-gap opening^7, 10, 16^, and carrier scattering^5, 9^, or enhancement of the spin-orbit interaction allowing for spin polarized transport at low magnetic fields^17^.

Depending on the physical dimensions, different types of corrugations can be defined^3^, such as graphene ripples, wrinkles, folds, or crumples. Graphene ripples^1, 18^ have a length/width aspect ratio close to 1 and a height <1 nm; they act as out-of-plane deformations stabilizing the 2D graphene layer. Graphene wrinkles have an aspect ratio larger than 1, reaching a length of up to a few micrometers, and they are formed when graphene experiences a uniaxial exterior force^3, 19^. Wrinkles are also formed in the area surrounding the defects in the graphene^8, 20–26^.

Graphene folds can be described as collapsed wrinkles. When the wrinkle exceeds the critical height, it is not standing perpendicular to the substrate; rather, it bends and collapses upon the fold formation^5^. Graphene crumples with dimensions that are more or less comparable to the dimensions of the wrinkles are dense isotropic corrugations in two or three directions. Crumples are formed when multidirectional forces are applied to graphene^2, 27, 28^, and crumpled graphene exhibits tunable wettability and transparency^28^. Based on experimental observations, crumpled and wrinkled graphene are both promising candidates for energy-related research (such as fuel cells and supercapacitors), which requires high conductivity, high surface area, thermal stability, and mechanical and chemical robustness^29, 30^.

Considering the application of corrugated graphene, it is essential to fully control the wrinkling^31^ and crumpling so the corrugated graphene will be beneficial for targeted graphene-based device fabrication. The wet transfer of graphene onto any substrate (flat or patterned) often leads to the creation of wrinkles and crumples^3, 32, 33^. Crumple formation is typically connected to the fast evaporation of the liquid used for the wet transfer. Formation of the specific graphene wrinkle networks can be done via the transfer of graphene onto patterned substrates^34–36^. Many approaches have been presented in the literature^31, 36, 37^, such as graphene transferred onto nanoparticle-decorated substrates, when parts of graphene are still in contact with the substrate^38–41^, or graphene transferred over arrays of pillars^42, 43^. It has been shown that transfer of graphene over substrates decorated with homogeneously distributed nanoparticles leads to the formation of wrinkles with specific density and surface/projected area^40^. Those systems demonstrated the tunability of the wrinkle network morphology and the graphene’s intrinsic properties by changing the distribution of the nanoparticles on the substrate^38, 40^.

Studies examining graphene-nanopillar systems have revealed that tuning of the pillar geometry (shape of the pillar apex, pillar height) should lead to the transformation of collapsed graphene into fully suspended graphene^43^, forming either a smooth or wrinkled graphene layer depending on the pillar density and geometry^42–46^. After reaching the critical pillar distance (minimizing the inter-pillar distances), all the large wrinkles that are formed are localized near/on the pillars, oriented in the direction of the symmetry axis of the pillar arrays. Finally, line defects in graphene has been found to introduce defined wrinkle and ripple networks^47^. As demonstrated in previous research, graphene corrugation is a highly complex issue with many degrees of freedom. Therefore, a rigorous analysis of the wrinkle patterns and the substrate topography should always precede the interpretation of the resulting graphene arrangement.

In this paper, we present an approach to prepare well-defined large arrays of graphene wrinkles with predictable geometry, and we report the existence of fully-suspended graphene on nanopillar arrays with inter-pillar distances up to 1 μm. In contrast to a previous study^43^, our graphene layers are self-supporting and do not form tent-like structures, which touch the substrate beneath the pillars. A thorough analysis of the wrinkling networks, primarily using Atomic Force Microscopy (AFM) and Raman Spectroscopy (RS), shows an unexpectedly large number of different wrinkles in terms of the preferred propagation direction and propagation length, despite the regularly-ordered hexagonal pillar arrays that locally support the graphene monolayer. These wrinkles can be categorized into two main types, and their origin explained by the presence or absence of distinct long features that are characteristic of chemical vapor deposition (CVD)-grown graphene: the grain boundaries and/or thin wrinkles originating in the graphene transfer.

## Result

### Basic sample characteristics

**Figure 1:**
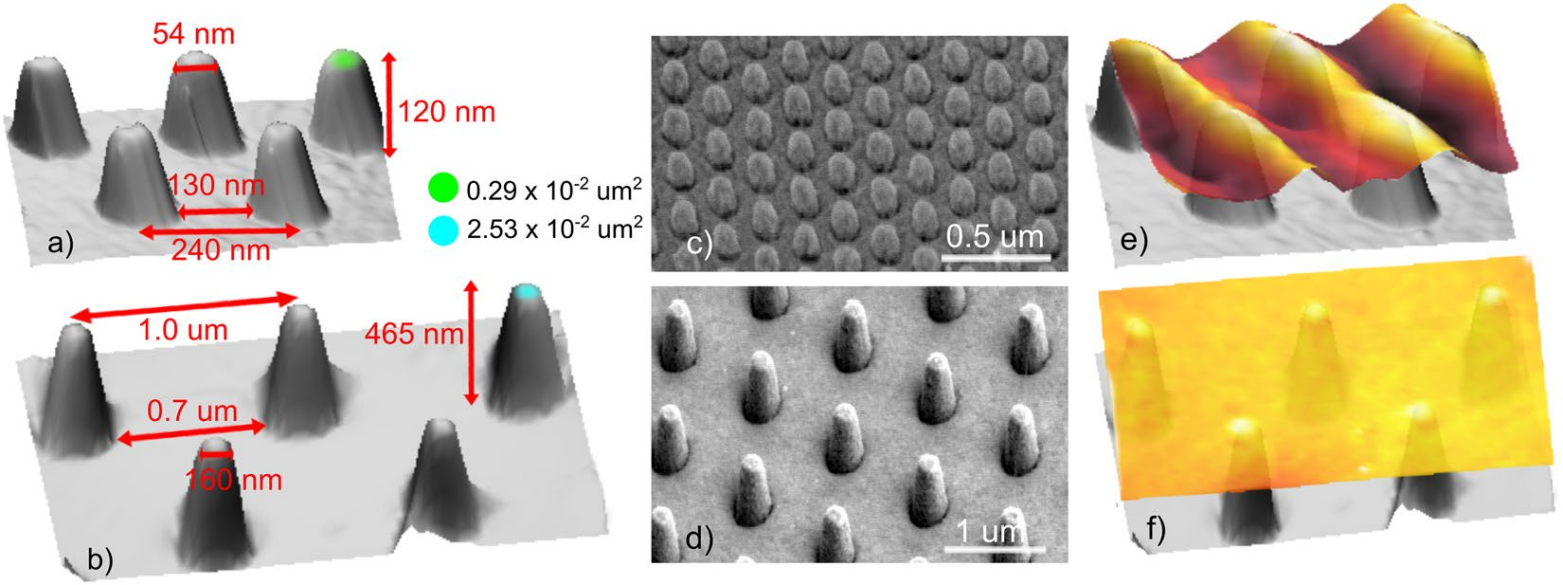
Illustration of the bare and graphene-covered nanopillar arrays. Three-dimensional (3D) AFM topography image of the nanopillars of (**a**) the GN@pillars_A and (**b**) the GN@pillars_B samples with the selected structural dimensions labelled. Green and blue spots correspond to the projection of the pillar cut in 10% of the pillar height. Scanning Electron Microscopy (SEM) images of the nanopillar arrays of (**c**) the GN@pillars_A and (**d**) the GN@pillars_B samples. Side-view of the 3D AFM topography illustration of (**e**) the GN@pillars_A sample with aligned wrinkles on the top of the pillars; and (**f**) the GN@pillars_B sample with the almost flat graphene layer on the top of the pillars.

We prepared model structures of suspended topographically-corrugated graphene by transferring the CVD-grown graphene monolayers onto the top of the nanopillar arrays that were fabricated using electron beam lithography on Si/SiO_2_ substrates. Two pillar-array geometries with graphene suspended from the substrate were made, each with a different nanopillar density, and, consequently, a different geometry and morphology of the transferred graphene layer. The used nanopillar arrays form a hexagonal (*hcp*) lattice (Fig. 1), their height, *h*_p_ reaches 50% of the inter-pillar distance, *l*_p-p_ (see Table 1 and Fig. 1 for the basic geometrical dimensions). Depending on the nanopillar density, the samples were labelled as the GN@pillars_A (18 nanopillars/μm^2^) and the GN@pillars_B (1 nanopillar/ μm^2^) samples.

**Table 1:** Parameters of the nanopillar A and B arrays, summarizing the number of pillars per 1 μm^2^, pillar height *h*_p_, radius of pillar tip curvature, *r*_A_, distances between two pillars measured between pillar centers *l*_p-p_, inter-pillar distance measured from the edges of the pillar bases *l*_int_, diameter of the pillar cross-section in 10% of the maximum pillar height, *d*_top_, diameter of the pillar base, *d*_base_, spherical cap surface area and substrate pillar coverage, *C*.

sample	no.pillars/μm^2^	*h*_p_ (nm)	*r*_A_ (nm)	*l*_p-p_ (nm)	*l*_int_ (nm)	*d*_top (_nm)	*d*_base_ (nm)	*S*_top_ × 10^−2^ (μm^2^)	*C* × 10^−2^ (μm^2^)
GN@pillars_A	18	119	27.5	240	130	54	111	0.29	2.78
GN@pillars_B	1	465	54.3	1000	700	159	228	2.53	4.08

**Figure 2:**
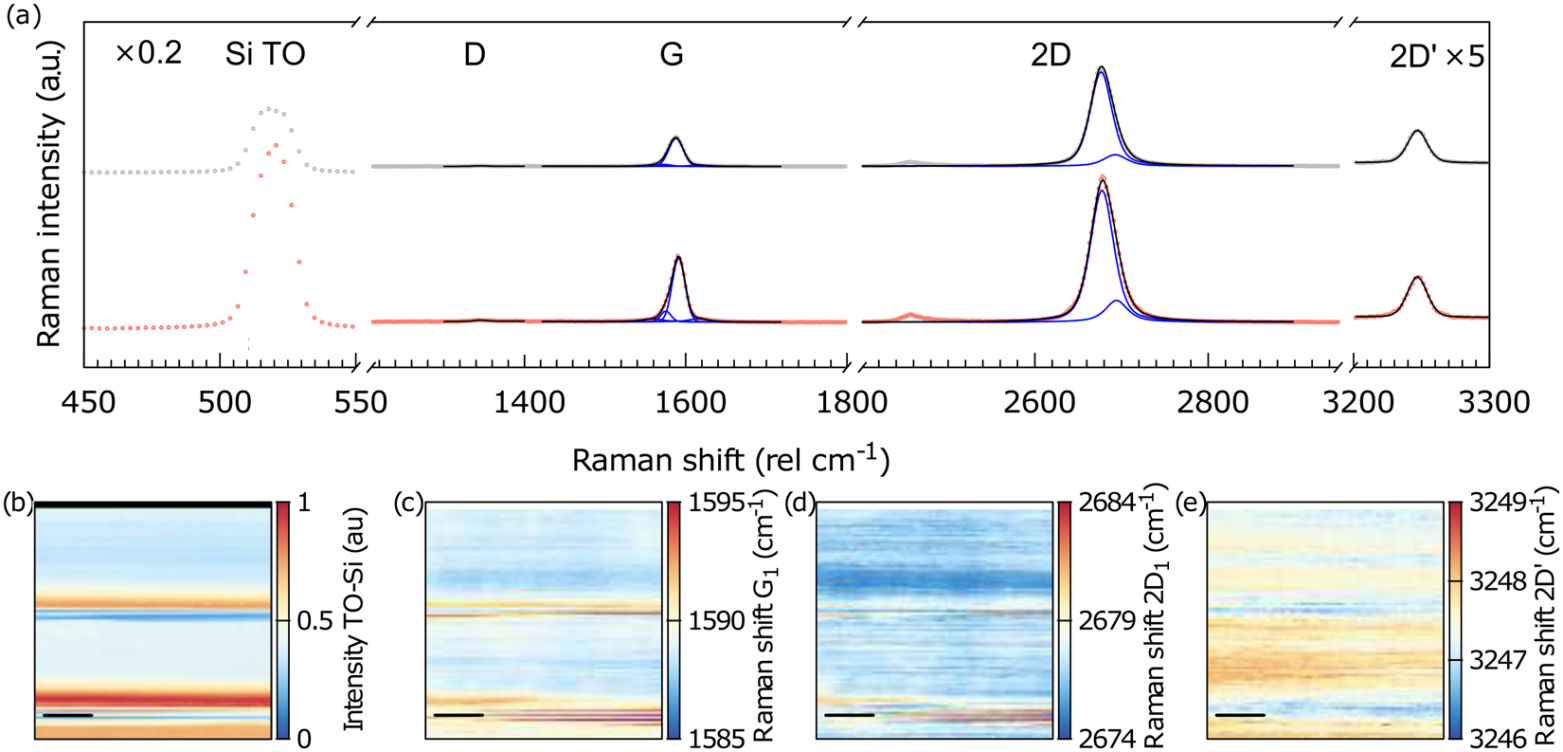
(**a**) Typical Raman spectra of the GN@pillars_A sample for regions with random wrinkled graphene (grey data points) and aligned wrinkles (red data points); (**b**) intensity map of the transverse optical (TO) phonon mode of Si at 520.2 cm^−1^. The clearly visible horizontal lines with enhanced intensity of the TO mode are attributed to the areas with wrinkled graphene (aligned area). It is important to note that, depending on the position of the laser spot, one Raman spectrum contains information about the suspended and supported graphene, and the different wrinkle areas due to the laser spot size (see Methods). The Raman shift of the G_1_ (**c**), 2D_1_ (**d**) and 2D’ (**e**) modes of graphene. The scale bar in all the maps represents 1 μm.

**Figure 3:**
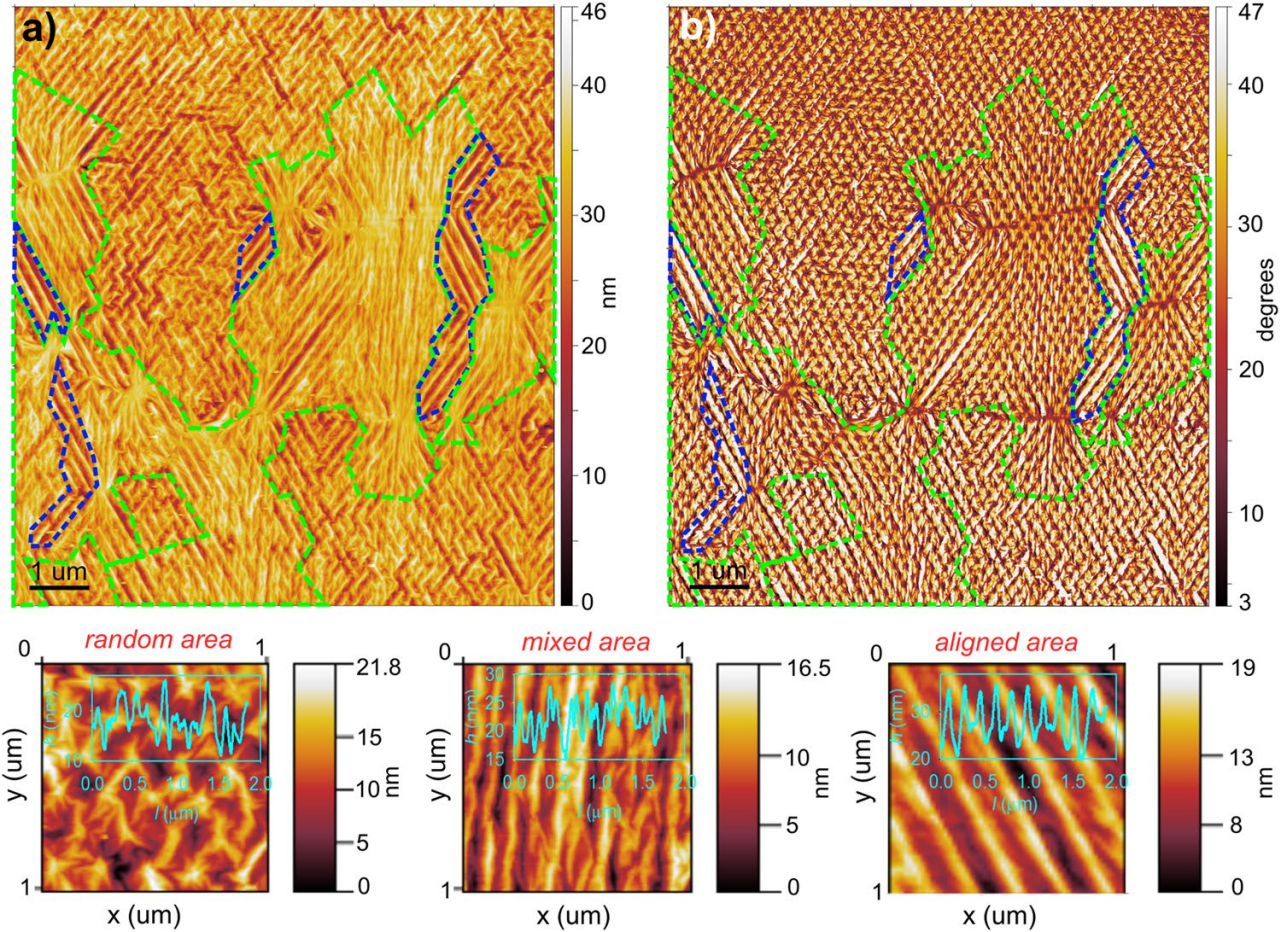
AFM image of the GN@pillars_A sample; (**a**) topography; (**b**) phase; visualizing the aligned and mixed areas in navy blue and light green dashed areas, respectively. Details of the wrinkled areas with different morphologies: random, mixed, and aligned areas with the typical profile-cross section in the insets. The random area is characterized by multiple minor wrinkles spreading away from the central pillar, and the main wrinkles propagating in two main directions forming an angle of ~60° (see the Supplementary information file for details). The mixed area is characterized by major wrinkles developing in the direction of the symmetry axis of the nanopillar array, with the second network of minor wrinkles propagating in the direction of the 2^nd^ NN, thereby connecting the major wrinkles. The aligned area is characterized by uniaxial propagation of the main wrinkles over very large distances in the direction of the symmetry axis of the pillar array, with a negligible amount of minor wrinkles propagating in the region between the major wrinkles.

Inspection of both samples by RS revealed no apparent D mode within the graphene layers, indicating the high quality of the graphene layer (see Figs 2 and S3), with an insignificant amount of defects. AFM demonstrated that the graphene layer is fully suspended from the substrate (see Figs 3 and S1), independently of the nanopillar density.

### Wrinkle networks

**Figure 4:**
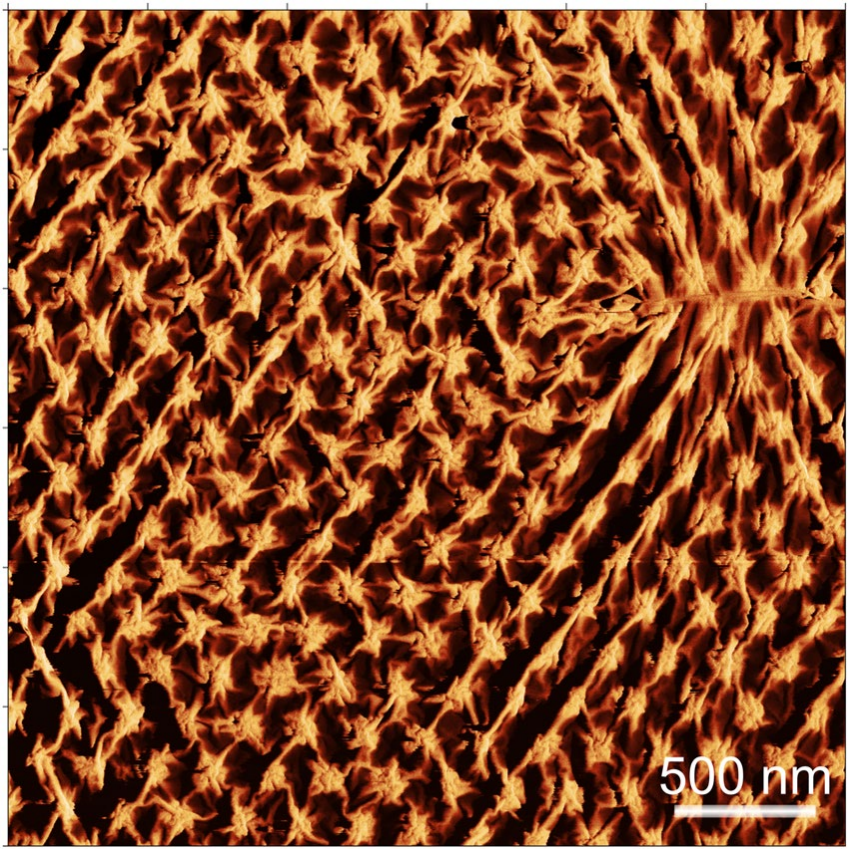
Visualization of the wrinkle location with respect to the nanopillar positions. The image was created as a subtraction of the AFM topography and phase images. The AFM topography image shows the complete wrinkle network, whereas the phase image visualizes both the topography corrugations (wrinkle tops, cracks in the graphene-grain boundaries) and the objects hidden beneath the graphene layer, which are in direct mechanical contact with the graphene (pillar tops). A combination of both images via image arithmetic allows for visualization of the topography and the mechanical perturbations of the scanned surface.

**Figure 5:**
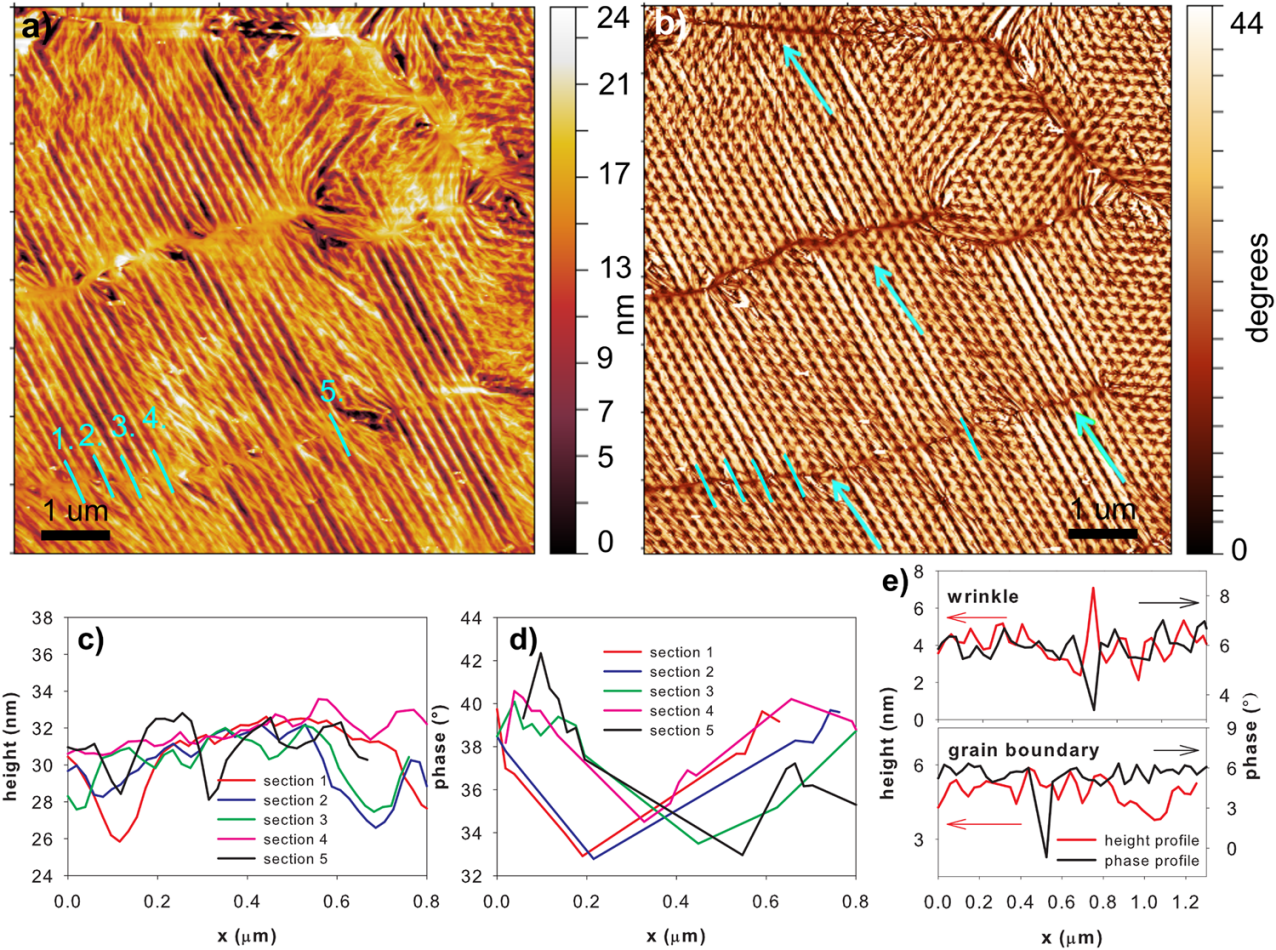
Formation of the aligned wrinkle networks induced by the graphene line interfaces (marked with green arrows); (**a**) AFM topography and (**b**) phase images of the GN@pillars_A sample; (**c**,**d**) show cross-sections of the height and phase images in the positions of green lines in (**a**,**b**). As seen, the graphene line interface is barely observable in the AFM topography (**a**) and the position of the interface cannot be determined from the cross-sections of the topography (**c**); (**e**) typical profile of the phase contrast for a wrinkled and flat grain boundary. There is no change in the height (topography) for the grain boundary.

As seen in Fig. 1 and Figs 3, 4 and 5, the graphene layer on the nanopillars is not flat; it forms a large amount of wrinkles. Moreover, the wrinkle networks created in our graphene layers are not completely homogeneous. Surprisingly, areas with different, very distinct wrinkle morphologies can been seen (Figs 3, 4 and 5 and S1) even though the supporting pillars are created in an ordered fashion. All the wrinkles are created on the top of the pillars (with the cross-section areas reaching 0.29 μm^−2^ and 2.53 μm^−2^ for the GN@pillars_A and the GN@pillars_B samples, respectively (Fig. 1), not in the inter-pillar regions, and the wrinkles are extend from the first or second nearest neighbor (NN) pillars (see Figs 3 and 4) up to large distances. The GN@pillars_A sample exhibits a complex wrinkle network, whereas the GN@pillars_B sample shows an almost flat graphene layer, with sparse topographical corrugations appearing at the edges of the gaps in the graphene layers. The complex structure of the GN@pillars_A sample is discussed in further detail in this paper (see following paragraphs), whereas the morphology of the flatter and smoother GN@pillar_B sample is described in the Supplementary information file.

Depending on the wrinkle propagation length, two types of wrinkles were identified in the GN@pillars_A sample: minor wrinkles and major wrinkles. The minor wrinkles propagate in short-range distances (no farther than to the 2^nd^ NN), and the major wrinkles extend over multiple inter-nanopillar lengths (see Figs 3 and 4, and Figure S6a).

The minor and major wrinkles do not propagate randomly over the nanopillar array; rather, they form well-defined wrinkle networks. Careful examination of the AFM images (see the example in Fig. 3) shows that the entire morphology of the graphene layer is formed by three characteristic wrinkle areas, defined by specific wrinkle alignment.

Those characteristic wrinkle areas with different morphologies are the (i) aligned, (ii) mixed and (iii) random areas, depending on the wrinkle type, the direction of the wrinkle propagation, and the symmetry of the wrinkle network. Specification of the individual wrinkle area morphology is summarized in Table 2. The main difference between the aligned areas and the random areas is the length of the wrinkles and their propagation direction. Wrinkles in the aligned areas are separated equidistantly, and they feature long-range propagation (reaching tens of micrometers), whereas wrinkles in the random areas are characterized by a short-range propagation length of about 2.5 *l*_p-p_ (Table 2). The so-called mixed area exhibits properties of both the aligned and random areas, as the major wrinkles in the mixed area propagate over a large distance, whereas the minor wrinkles form networks of short wrinkles propagating between the major wrinkles (see Fig. 3).

**Table 2:** Parameters of the wrinkled graphene layer of the GN@pillar_A sample.

A_GN_/μm^2^ (%)	Area type	*A*/*A*_GN_ (%)	*S*_*GN*_ */um* ^2^ *(%*)	*S*_*cont*_ */um* ^2^ *(%)*	*l*_*wr*_ *(l*_*p-p*_ *)*	*R*_tm_ (nm)
99	Random area	24	103	5.1	2.5	10.6
	Aligned area	51	101	5.2	5.0 -35.0	11.0
	Mixed area	25	113	4.6	1.7	5.0

Percentage of the surface area of the sample covered with graphene, *A*_GN_, number of different types of the graphene areas within individual samples with the list of individual area types, surface area attributed to the different area types, *A*/*A*_GN_, surface area of wrinkled graphene, *S*_GN_, calculated contact area of the graphene and the nanopillar, *S*_cont_, typical wrinkle length, *l*_wr_ and median peak-to-peak amplitude for different area types, *R*_tm_, corresponding to the slack of the graphene between individual wrinkles.

The median peak-to-peak amplitude of the wrinkled graphene layer, *R*_tm_, (corresponding to the peak-to-valley distance of the wrinkle network defined in Equation 1 and the Supplementary Information File) is almost the same for the random and aligned areas (*R*_tm_ ~ 11 nm), and it is two-times smaller for the mixed area (*R*_tm_ ~ 5 nm).

The aligned and mixed areas are formed in the proximity of specific line boundaries, which are visible as thin dark lines on the AFM phase images (see Figs 3b, S7–S10). The random areas are found exclusively in the line-boundary-free regions. We refer to the line boundaries as the graphene line interfaces; this will be discussed later on in this paper when we also discuss their origin.

The morphology of the wrinkle networks is completely changed when the nanopillar array dimensions are increased four-times (the GN@pillar_B sample). The graphene layer is then almost smooth, with scarce wrinkles created near the edges of the graphene sheet. The formed wrinkles do not propagate farther than 3 *l*_p-p_ away from the graphene edge in the perpendicular direction; hence, the central parts of the sheets that are larger than 6 × 6 (*l*_p-p_)^2^ are completely wrinkle-free. For details, please see Table S1 and Figure S1.

### Strain analysis

We used RS to examine the graphene layer quality, and to also investigate the strain, analyzing the G, 2D, and 2D’ modes^38, 48^. In analogy with the system of wrinkled graphene on particle-decorated Si/SiO_2_ substrate^38^, an elaborate fitting of the G and 2D modes was conducted to analyze separately the graphene on the pillars (G_2_ and 2D_2_ components) and between the pillars (i.e., suspended; G_1_ and 2D_1_ components; see Methods for details). In principle, it is necessary to distinguish between the aligned and non-aligned wrinkle networks of the graphene in the GN@pillars_A sample. We observed that the aligned areas containing long wrinkles are clearly recognized by the RS, forming the lines with enhanced intensity on the Raman maps (Fig. 2). Both of the studied samples (GN@pillars_A and GN@pillars_B) are almost defect-free, as the graphene D mode associated with the presence of defects is barely seen (Figs 2b and S3).

As seen in Fig. 2, the captured spectra of the aligned and random areas are not similar. A significant difference can be observed between the G mode of both areas, whereas the 2D mode of both areas is comparable. The G mode of the random area is rather symmetric, whereas the aligned area clearly exhibits an asymmetric G mode. We fit this asymmetry using one extra peak function (G3) that accounts for the long wrinkles (Figure S16). Furthermore, the width of the G mode is smaller for the graphene aligned area in comparison to the random area (see Supplementary for details).

As can be deduced from the Raman maps (Fig. 2d–f), the Raman shift of the G, 2D, and 2D’ modes is fairly homogeneous, except for the proximity of the aligned wrinkle area. Although a large strain within the graphene on the pillars would be expected^37^, the variations that are observable within the Raman 2D and 2D’ maps are relatively small. As shown in Fig. 2f, the difference between the minimum and maximum Raman shift of the 2D’ mode is about 3 cm^−1^, corresponding to a variation in the strain of only 0.1% (assuming that the upper bound presence of uniaxial strain corresponds to ∼21 cm^−1^/1%^48^). Note that the strain determined for the GN@pillars_B sample is higher in several regions of the graphene layer (see the Supplementary information file) in comparison to the GN@pillars_A sample.

The strained graphene areas were also examined by mapping the mechanical properties^49^ using AFM, which is capable of obtaining higher resolution than RS (see Figures S10 and S11). The mechanical deformation (the quantity represented as the displacement of the individual elements forming the object^49^) should be lower for the strained areas in comparison to the mechanical deformation of the unstrained graphene layer, as is known from the standard mechanics of materials^49^. Hence, the relative strain can be estimated measuring the mechanical deformation of the graphene layer (Figures S10 and S11). Indeed, we can observe that the measured mechanical deformation of the graphene is lower for the inter-pillar regions with the free-standing graphene than for the supported graphene on individual nanopillars (Figures S10 and S11). This can be explained either by different strain in the free-standing and supported regions^37^ or by probing of the substrate in the supported region. Moreover, in general, the mechanical deformation of the graphene between the pillars is higher for a dense pillar array (GN@pillars_A) than for the arrays with low-pillar density (GN@pillars_B). This suggests that the graphene in the GN@sample_B sample is stiffer and better attached to the pillars.

## Discussion

Generally, one would expect full delamination of the graphene from the flat part of the substrate decorated with pillar arrays with interpillar distances reaching 240 nm (the GN@pillars_A sample);^42, 43^ however, this was observed even for the 1 μm pillar separation length (the GN@pillars_B sample). Nevertheless, this observation is also in contrast to the findings reported by Reserbat-Plantey *et al*.^43^, which demonstrated a collapse of the graphene layer and the formation of a tent-like structure for the pillar arrays with an *l*_p-p_ of approximately 1 μm. A possible explanation for the self-supporting nature of the graphene layer in the case of the GN@pillar_B sample can be found in the mechanism used to prepare the sample^50^. We assume that the key factors for preventing the graphene from gliding down the pillars and, subsequently, adhering to the substrate, are the solvents that are used, the polymer that is used for the wet transfer^51–55^, and the strength of adhesion of graphene to the nanopillars.

It is important to discuss the origin of the most interesting issue addressed in this paper, which is the formation of the equidistantly separated parallel aligned wrinkles. We observed that the aligned and mixed areas of the wrinkles are formed on the graphene line interfaces, which are visible on the phase images as the dark thin lines (Figs 3, 4 and 5). In general, the aligned areas are located near the central interface parts if the interface length exceeds 6 *l*_p-p_, whereas the mixed areas are found near the interface edges. On the other hand, the interface-free graphene is characterized by short-range propagation of the wrinkles (the random wrinkle area). However, the formation of the wrinkle networks with predictable hierarchy on the line defects is not surprising.

As was suggested by several theoretical papers^8, 26, 56–58^ and demonstrated experimentally for the grain boundaries^47^, presence of any constraint in a thin sheet (constraint means 2D layer boundary such as a line defect represented by the graphene grain boundary or edge of the membrane) leads to the formation of the wrinkle networks with a predictable hierarchy. In general, specific mechanical stimulation^57^ and any confinement of the 2D membrane allows artificial control over wrinkle formation, resulting in a universal self-similar hierarchy of wrinkles^56, 59^. This was proven for strained graphene^32^, hanged fabric^56^, and graphene grain boundaries/line defects formed by pentagons and distorted hexagons^47, 58^. Origin of folding and wrinkling on the constraint is based on the competition of the stretching and bending of the constrained membrane^56^, because the bending rigidity of thin membrane is smaller than the stretching rigidity and membrane tends to reduce the elastic energy via formation of folds or wrinkles^59, 60^. The periodic wrinkling of thin membrane in the direction perpendicular to the constraint represents the equilibrium state of the constrained membrane, with the wrinkle wavelength increasing as λ~*x* ^m^, where *x* is the distance from the constraint (boundary) and *m* = 1/2 for graphene sheet^56^.

This implies that the existence of graphene line interfaces is critical for the formation of aligned wrinkle areas on our pre-patterned nanopillar substrates. The explanation of the origin of these graphene line interfaces is based on the analysis of the AFM phase images, correlated with the topography images.

Phase imaging and direct measurement of the phase shift, which, in general, is related to the local energy dissipation of the surface, enables visualization of the regions with different material properties. Moreover, the phase images of the graphene layers examined in this present study simultaneously visualize the topography corrugations and the objects hidden beneath the graphene layer (Figs 3b, 4 and 5, and S1b, S7–S9), allowing localization of the wrinkles with respect to the pillar positions. It is also possible to track the graphene grain boundaries using AFM phase imaging, as they exhibit chemical, electrical, and mechanical properties^61^ that are different from the rest of the graphene layer.

The wrinkles, the pillar tops, and the graphene line interfaces are manifested in the phase images as dark areas with low-phase values (see Figs 3b and 5b, and S1b). Several graphene line interfaces are also observable on the AFM topography images as thin wrinkles lacking a particular orientation with respect to the symmetry axis of the underlying pillar arrays. However, the rest of the low-phase interfaces are barely visible; the height of the generated wrinkles exceeds the height of the graphene line interfaces. Thus, we attribute the graphene line interfaces to two sources: (i) the thin, long non-oriented wrinkles that are formed due to the growth or transfer of the graphene^25, 62^ (we call them further transfer-wrinkles) and (ii) the graphene grain boundaries (marked with green arrows in the selected images)^47^. The transfer-wrinkles (i) are visible both on the topography and phase images, whereas the graphene grain boundaries (ii) are observable only on the phase images. To prove this presumption directly, we examined the graphene transferred onto the Si/SiO_2_ substrate using the same transfer method (see Figures S7–S9 in the Supplementary information file). In this case, the height variations are much smaller than they are in the pillar-supported area, which allows an unambiguous assignment of the wrinkles and the grain boundaries via the phase contrast, correlated with the AFM topography. As seen in Figs 3 and 5, the presence of the separated line interfaces is crucial for the formation of the aligned wrinkles. In our case, the regularly nanometer-sized and -spaced corrugations that spread away from the graphene grain boundaries^47, 58^ are transformed by the nanopillars into aligned wrinkles with larger wavelength/amplitude. Existence of these large arrays of equidistantly separated aligned long wrinkles forming on the grain boundaries of graphene sheet, which represents constraints in the thin membrane is expectable. As we have already discussed, the presence of any constraint in the thin membrane results in the spontaneous creation of long wrinkles. If a periodic boundary condition with the characteristic wavelength *λ*_boundary_ is introduced at the constraint (boundary), the initial wavelength of the wrinkles emerging at the boundary corresponds to *λ*_boundary_. In our case, *λ*_boundary_ is equal to the interpillar distance of our pillar array. The difference in the wrinkle propagation in a free thin membrane and our system is that the wrinkles are not propagating as λ~*x* ^m^ over their whole propagation length in our aligned wrinkle arrays, but they follow the symmetry of the pre-patterned substrate, hence the inter-wrinkle distance is kept equal to interpillar distance *l*_p-p_. Moreover, if we assume that the wavelength of a long wrinkle follows the λ~*x* ^m^ dependence at least between two nearest pillars, the estimated increase of the λ along the distance to the nearest pillar (*x* = *l*_p-p = _240 nm, thus Δλ ~ 15.5 nm) is smaller than pillar diameter (27.5 nm). When the next pillar is reached, the wavelength is ‘reset’. This implies that the first order wrinkle will always propagate to the nearest-neighbor (NN) pillar in the wrinkle propagation direction and does not pass between the pillars. Therefore, it is not surprising that the long parallel equidistantly separated wrinkles are formed on the grain boundaries and that they take on the dimensions of our nanopillar array. Furthermore, the particular orientation of the long wrinkle with respect to the grain boundary is given only by the mutual orientation of the nanopillar array and the grain boundary.

It remains to answer the origin of the wrinkle shapes found in the random areas, where the short-range wrinkles are present. We have not observed any boundary or constraint in the random areas, consequently formation of the long wrinkles with λ~*x* ^m^ is not expected. The longest formed wrinkles exceed in maximum to the 1^st^ NN pillar, if their length corresponds at least to the *l*_p-p_. If shorter wrinkles are created on the individual pillars (*l*_wr_ < *l*_p-p_ = 240 nm), the two individual wrinkles emerging from two individual NN pillars form the so called ‘avoiding pairs’ of wrinkles, preventing their joining. The observation of ‘avoiding pairs’ of wrinkles in the random areas corresponds well with the simulations of Budrikis *et al*.^63^, who simulated the conditions under which two wrinkles formed on two separated objects can join or form the ‘avoiding pair’. Comparing the geometry of our system with the simulations, the length of the wrinkle created on individual pillars with no external anisotropic effects should reach almost 140 nm, and prevents joining with the wrinkle formed on the NN pillar (following the notation in the manuscript^63^, Fig. 2, *X/Y* = 1, *R* = 240 nm, ξ ~ 140 nm, *u/v* ~ 0.2).

Even though there is a non-zero probability that the two wrinkles join, the probability of creating an ordered array in such a way is negligible, as can be seen, e.g. in Figs 3 and 4.

In addition, the strain determined for both samples corresponds qualitatively with the different sample morphologies. The GN@pillars_B sample exhibits larger strain than the dense system of pillars (the GN@pillars_A sample) when comparing the free-standing regions, which is confirmed both by the RS and the lower measured mechanical deformation of the more strained layer (Figures S10 and S11). In the GN@pillars_A sample, we can assume that the strain is released through the wrinkle formation; in the GN@pillars_B sample, flattening of the layer is observed. Larger stretching of the layer for the GN@pillars_B sample (compared to the GN@pillars_A sample) resulting in almost flat-like nature of graphene transferred over the large pillar arrays is not surprising. If we assume that the pillar top surface area (*S*_top_) is more than ten-times larger for the pillars in the GN@pillars_B sample than for the GN@pillars_A sample (2.53 vs. 0.29 × 10^−2^ μm^2^), the graphene/pillar contact region, hence its adhesion, is much larger for the GN@pillars_B sample. Consequently, the graphene is prevented from slight gliding down from pillars and forming of wrinkles, as the graphene membrane can be stretched more efficiently between the large pillars. This wasindirectly confirmed by larger strain for the GN@pillars_B sample than for the GN@pillars_A sample reaching 0.1%; and smaller median deformation for the GN@pillars_B sample (8 nm) than for the GN@pillars_A sample (12 nm), both together proving better adhesion of graphene to the large pillars. Therefore, the GN@pillar_B sample is proposed to be a candidate for strain-induced band-gap engineering^37, 64^ (using pillar arrays with specifically-designed symmetry) and the creation of pseudomagnetic gauge fields^65–67^.

In conclusion, we prepared a model system of fully-suspended, self-supporting graphene on nanopillar arrays with inter-pillar distances up to 1 μm. We showed that, for the dense nanopillar array with inter-pillar distances ~240 nm, topographically-corrugated graphene formed three well-defined types of wrinkle networks with a predictable wrinkle hierarchy. After observing equidistantly separated parallel aligned graphene wrinkles on the graphene line interfaces (thin wrinkles and graphene grain boundaries), we discussed how the geometry of the wrinkle networks can be tuned up. If the position of the graphene line interfaces is controlled, one can prepare large regions of aligned wrinkles propagating over large distances with the inter-wrinkle spacing corresponding to the inter-pillar distances. By demonstrating that it was possible to manipulate the wrinkle network morphology and that the present line defects allowed 3D patterning of 2D material, we were able to prove existence of the self-supporting nature of the graphene layer with periodic wrinkle patterns.

## Method

### Sample preparation

Two types of samples of the graphene transferred onto the top of Si/SiO_2_ nanopillar arrays with different geometries were prepared. The nanopillar arrays were made by electron beam lithography, evaporation and lift-off-technique to create the hard mask pattern and subsequent Inductively Coupled Plasma (ICP)-etching. The wafer layout consists of 60 numbered chips with a size of 5 × 10 mm². Each chip contains a 1 × 1 mm²-field of nanopillar gratings. Hexagonal nanopillar gratings with different periods (240 and 1000 nm) were fabricated.

The fabrication starts on wafer-level by using oxidized 4″ Si wafers (125 nm SiO_2_-thickness for 240 nm gratings, 365 nm oxide for 1000 nm pitch gratings). The pre-cleaned Si wafers were coated with a two-layer lift-off -resist (150 nm AR6200.09 on 70 nm ARP617.03, both resists are from the Allresist Berlin GmbH) and additionally covered with a 10 nm thick gold conduction layer. The e-beam exposure was performed using the shaped beam writer SB350 OS (50 keV, Vistec Electron Beam GmbH) at an electron dose of 750 to 2000 µC/cm² to vary the structure sizes. The resist was developed for 60 s in AR600–546 and 30 s in MIBK:IPA = 1:3, always rinsed in IPA. Afterwards 40 nm of aluminium were deposited on top of the wafer by thermal evaporation under normal incidence. The lift-off process was carried out in a 10:1- mixture of 2-methoxyethanol and acetone. The lifted aluminium patterns serve as hard mask for etching into the oxide film. By using an ICP etching process with CHF_3_/SF_6_ and with some over-etching into the Si-wafer, needle-shaped nanopillar patterns are formed (Figure S15). After the wet chemical removing of the aluminium mask the wafer was covered with photo resist to avoid contaminations during the cutting process for the chip separation. After cutting and before use in experiments the chip-surface was cleaned by removing the resist using an acetone and isopropanol bath.

A graphene was prepared using chemical vapor deposition (CVD) growth on the Copper (Cu) foil, as described previously in^68^. The polycrystalline Cu foil was heated to 1000 °C and annealed for 20 min under a flow of 50 standard cubic centimeters per minute (sccm) H_2_. The Cu foil was exposed to 30 sccm CH_4_ for 20 min, after which the Cu foil was cooled to room temperature. The as-grown graphene was subsequently transferred to the substrate with the pillars using nitro-cellulose (collodion solution for microscopy, 2% in amyl acetate, Sigma Aldrich 09817), according to procedures reported previously^69^. The nitro-cellulose was removed from the graphene by annealing the sample at 180 °C for 30 min in air.

### Sample characterization

The prepared samples were characterized using complementary methods, including Field-Emission Scanning Electron Microscopy (FESEM), Atomic Force Microscopy (AFM), and Raman Spectroscopy (RS). FESEM S-4800 (Hitachi) was used for direct visualization of the prepared nanopillar arrays as well as the final samples of the nanopillars covered with graphene, using acceleration voltage of 1–5 kV.

The Raman spectra were obtained using a WITec Alpha300R spectrometer equipped with a piezo stage. The Raman spectral maps were measured with 2.33-eV (532-nm) laser excitation, a laser power of approximately 1 mW, and a grating of 600 lines/mm to obtain the entire spectrum from the Si TO mode to the 2D’ mode at once and lateral steps of 50 nm in both directions. The laser was focused on the sample with a 100× objective to a spot with a diameter of approximately 500 nm. All the spectra were fitted with pseudo-Voigt functions to obtain the Raman shift ω, full width at half-maximum (FWHM), intensity (I), and the fraction of the peak that is Lorentzian. To account for the wrinkles in graphene and the graphene that is in contact/not in contact with a pillar, the G and 2D modes were fitted with 4 and 2 pseudo-Voigt functions, respectively; G_1_ and 2D_1_ components account for the wrinkled suspended graphene, the other pseudo-Voigt functions (G_2_, 2D_2_) account for the graphene that is in contact with the Si/SiO_2_ pillars^38^. In the aligned wrinkle areas, an extra peak function (G_3_) accounts for the long wrinkles. Finally, the fourth pseudo-Voigt function of the G mode accounts for the D’ mode (Figure S16, Supplementary). Because the intensity of the D and 2D’ modes is very low, these modes were only fitted with a single pseudo-Voigt peak function. This analysis follows the procedure brought forward for highly wrinkled graphene partially suspended between nanoparticles on Si/SiO_2_ substrate, where a clear correlation between the intensity of the individual components and the ratio of suspended/supported graphene was shown^38^.

The tapping mode AFM images were captured at ambient conditions using a Multimode V microscope (Veeco) equipped with a JV scanner, with a resolution of 1024 lines. Fresh RFESP probes (*k* = 3 N/m, f_0_ = 75 kHz, nominal tip radius = 8 nm; Bruker, Inc.) were used for imaging the individual samples, preserving the comparable wear of the tip. The scan rate (0.6 Hz) and the amplitude setpoint were chosen in order to the minimize forces acting on the graphene layer. The images were captured with a maximum image size of 10 × 10 um^2^. High quality images were processed in the standard way using Gwyddion software^70^, applying line-by-line 1^st^ order levelling and scar removal. The images were further analyzed using image analysis, 2D Fast Fourier Transform (2D﻿ FFT) analysis and filtering, image arithmetic and grain analysis in Gwyddion software, and analysis of profile height distribution implemented using custom-made software. This study used 2D FFT analysis to determine the periodicity of the nanopillar pattern, the inter-pillar distances, and the preferential propagation of the graphene wrinkles. We used 2D FFT filtering to separate the long-range and short-range topography components and to separately visualize the long wrinkles and fine wrinkle structure. Image arithmetic was applied to the topography and phase images to obtain a combined visualization and evaluation of correlated morphological and phase features. Grain analysis was used to evaluate the surface coverage by the nanopillars and to determine the cross-section areas in the defined height and contact areas between the graphene and the nanopillars. Profile height distribution was analyzed to determine the median peak-to-valley distance, *R*_tm_, of the graphene layer topography profile that was attributed to the hanging of the graphene layer. The topography AFM image was cut into individual lines using home-built procedures, in order to identify all the minima and maxima attributed to the highest parts of the wrinkles and the lowest parts in the inter-wrinkle valleys. Then, the median difference of all maxima and minima was calculated as:

1 $${R}_{{\rm{tm}}}={p}_{\mu }-{v}_{\mu }$$

where *p*_*μ*_ is the median wrinkle height and *v*_*μ*_ is the median valley depth, calculated over all the lines in the image. For details on the procedure, see the Supplementary Information File (Figure S14).

Peak Force quantitative nano-mechanical mapping (PeakForceQNM) was done using the Dimension Icon Atomic Force Microscope (Bruker, Inc.); this complementary method was used to reveal the origin of the phase contrast. The images were captured using a SCANASYST-AIR probe (*k* = 0.4 N/m, *f*_0_ = 70 kHz, nominal tip radius = 2 nm; Bruker, Inc.). The captured topography, adhesion, and deformation images were processed using Gwyddion software, as described in the previous paragraph. The adhesion and deformation images of the graphene were qualitatively compared with the phase images captured in tapping mode.

## Electronic supplementary material

Supplementary Information File
