# Supplementary material for: Mastering the Wrinkling of Self-supported Graphene
Source: Sci Rep. 2017 Aug 30;7:10003. doi: 10.1038/s41598-017-10153-z (PMC5577149; doi:10.1038/s41598-017-10153-z)
Supplement: Supplementary file 1 — Supplementary Information File [file 41598_2017_10153_MOESM1_ESM.pdf]

Supplementary information file for

Mastering the Wrinkling of Self-supported Graphene

Barbara Pacakova,[1,2], Tim Verhagen[1], Milan Bousa[2,3], Uwe Hübner[4], Jana Vejpravova,[1,5],

Martin Kalbac[2], Otakar Frank[2#]

[1]Institute of Physics of the CAS, v.v.i, Na Slovance 2, 182 21, Prague 8, Czech Republic
[2]J. Heyrovsky Institute of Physical Chemistry of the CAS, v.v.i., Dolejskova 3, 182 23, Prague 8, Czech Republic
[3] Department of Inorganic Chemistry, Faculty of Science, Charles University, Albertov 6, 128 43, Prague 2, Czech Republic
[4]Leibniz Institute of Photonic Technology (IPHT), PO. Box 100239, D-07702 Jena, Germany
[5] Department of Condensed Matter Physics, Faculty of Mathematics and Physics, Charles University, V Holesovickach 2, 180 00 Prague 8, Czech Republic
# email: otakar.frank@jh-inst.cas.cz

Morphology of the GN@pillars_B sample

Figure S1 and S2 show typical AFM image of the GN@pillars_B sample. It is visible that topography of the graphene layer is almost smooth, compared to the GN@pillars_A sample.

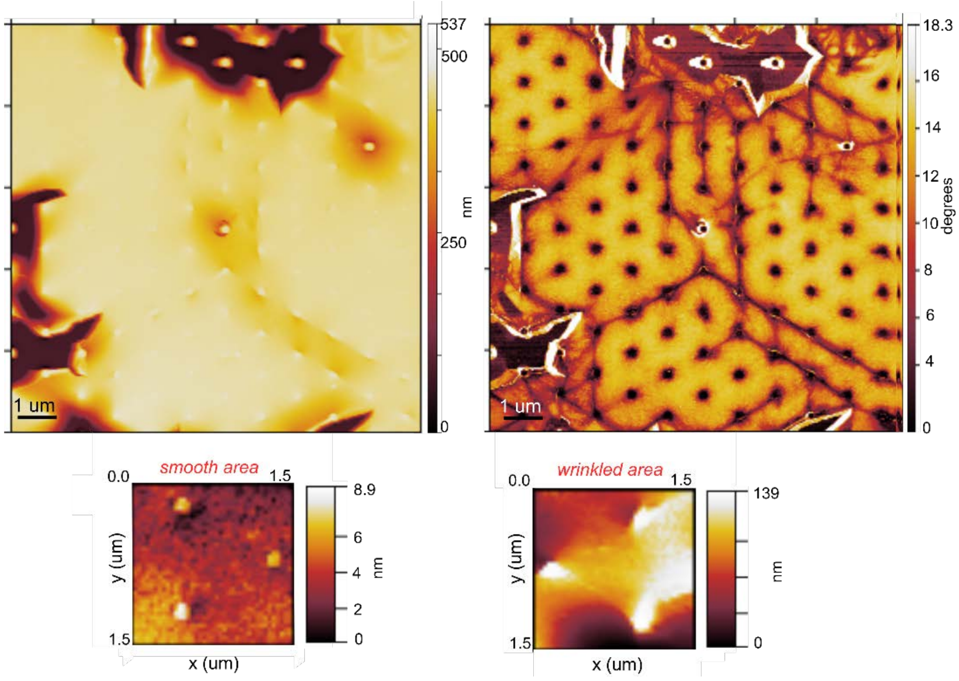

Figure S1. AFM image of the GN@pillars_B sample,topography (upper left) and phase (upper right). Details of wrinkled areas with different morphology are depicted in the lower part of the image – smooth and wrinkled area.

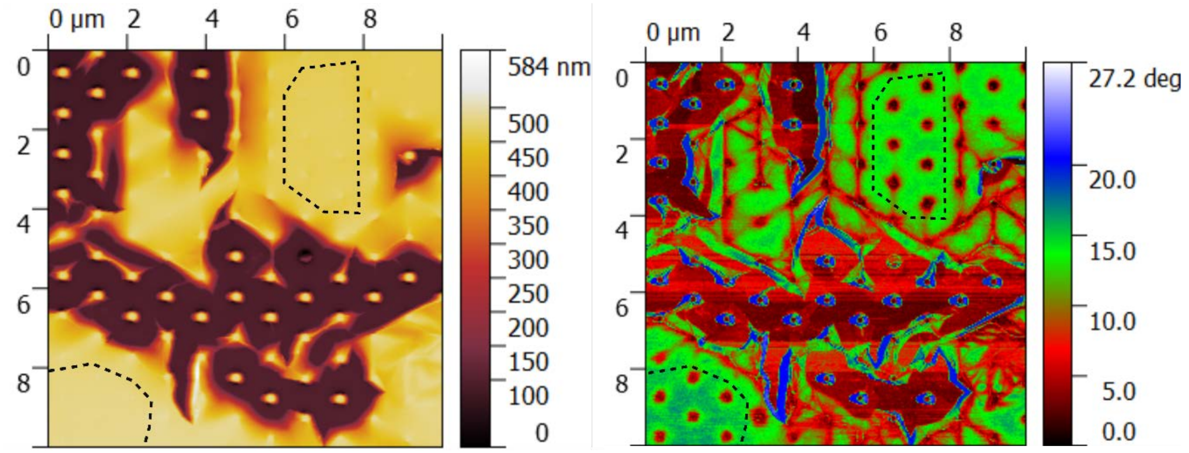

Figure S2. Demonstration of wrinkled edges of the graphene sheet for the GN@pillar_B sample, with almost smooth graphene in the central parts of graphene layers. Completely smooth graphene parts in the AFM topography (left) and phase (right) images are highlighted by black dashed lines.

Table S1. Parameters of wrinkled graphene sheet in the GN@pillars_B sample. Percentage of surface area of the sample covered with graphene, A_{GN}, number of different types of the graphene areas within individual samples with the list of individual area types, surface area attributed to the different area types, A/A_{GN}, surface area of graphene, S_{GN}, calculated contact area of graphene and nanopillar, S_{cont}, typical wrinkle length, l_{wr} and median peak to valley distance for different area types, R_{tm}, corresponding to the slack of graphene between individual wrinkles.

*wrinkles formed in the direction perpendicular to the graphene hole edge

sample	$A_{GN}/\mu m^2$ (%)	No. area types	Area type	A/A_{GN} (%)	S_{GN}/um^2	S_{cont}/um^2(%)	$l_{wr}(l_{p-p})$	R_{tm}(nm)
GN@pillars_B	59	2	smooth	80	1.00	2.5	x	1 -- 2
			wrinkled	20	x	X	3*	7 -- 25

Raman spectroscopy of the GN@nanopillars_B sample

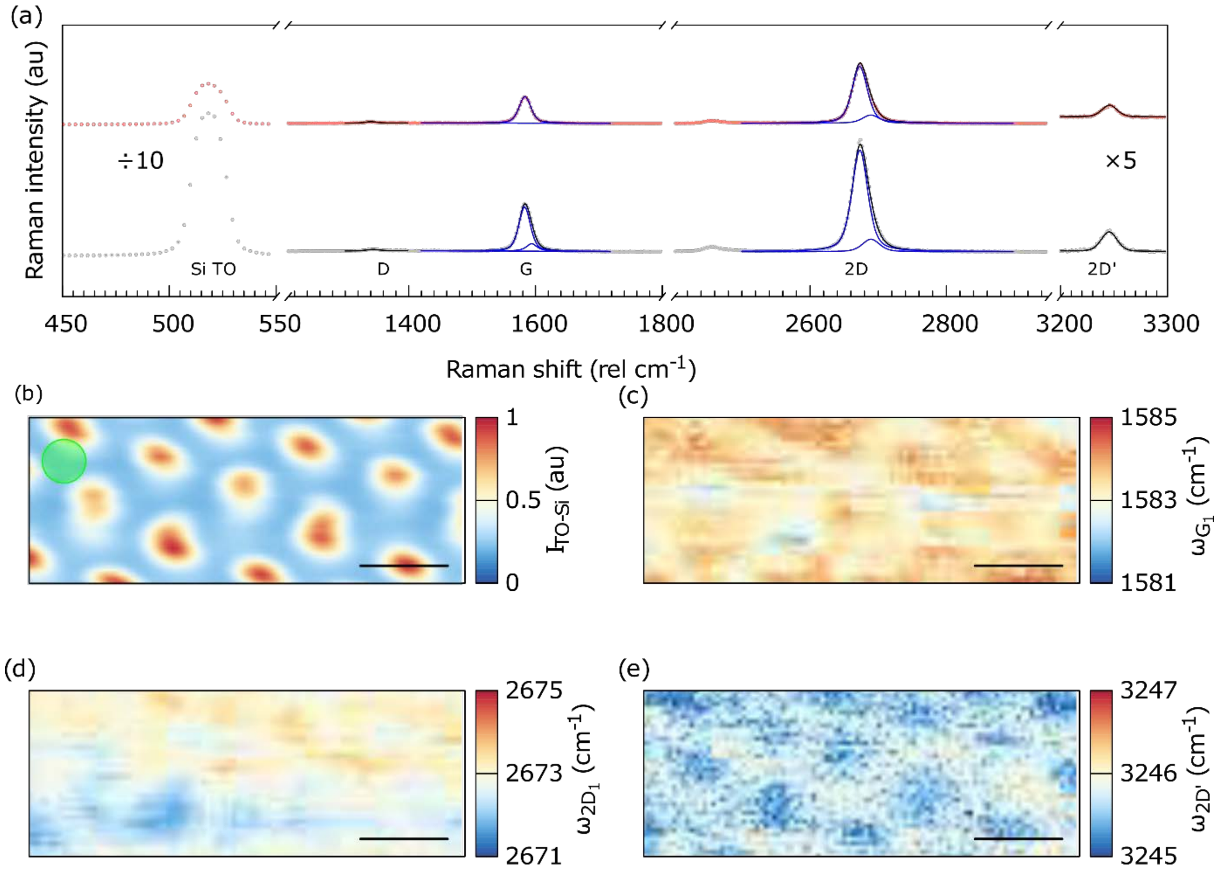

Figure S3. (a) Typical Raman spectra of the GN@pillars_B sample for region of freestanding graphene (red data points) and graphene supported by the Si/SiO_2 nanopillars (grey data points). (b) Intensity map of the transverse optical (TO) phonon mode of Si together with the typical size of the laser spot (green dot). It has to be noted that depending on the position of the laser spot, one Raman spectrum contains information both about the suspended and supported graphene due to the finite laser spot size. The Raman shift of the G_1 (c), $2D_1$ (d) and 2D' (e) modes of graphene. Scale bar in all maps represents 1 μm.

Optically visible large wrinkles

Figure S4 proves that large optically visible wrinkles (folds) are not the source of the aligned wrinkle formation, as they do not form continuous constraint boundary.

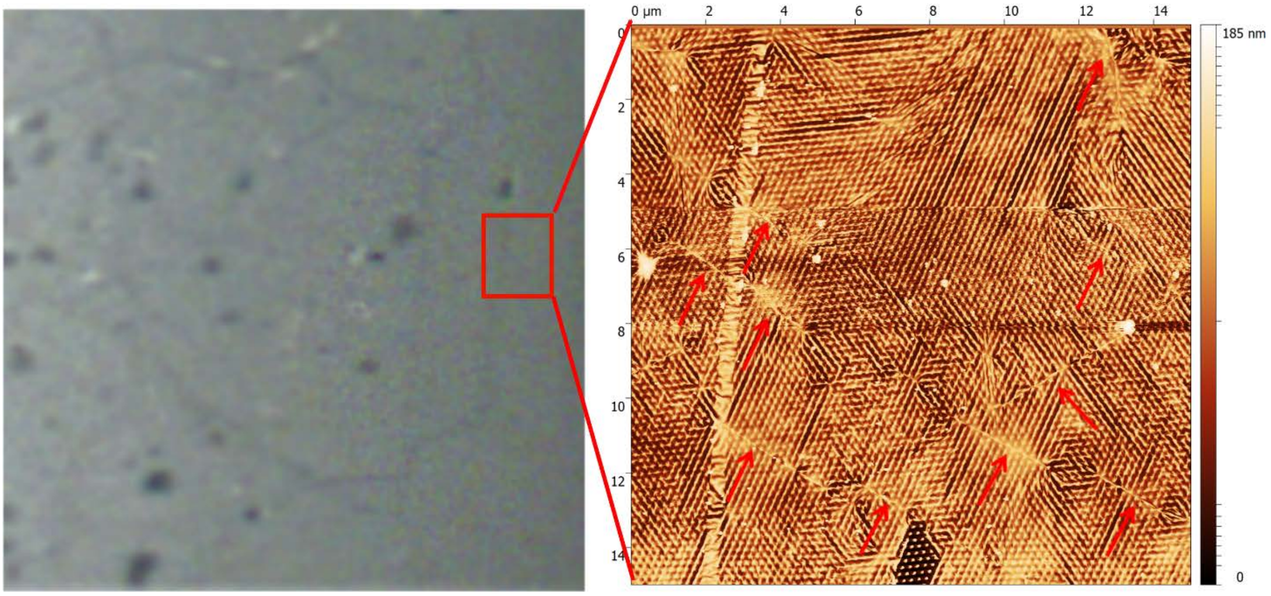

Figure S4. Visualisation of the optically visible wrinkle (left image) on AFM topography image (right). Left image shows typical optical pattern of graphene transferred on the nanopillar array, with thin wrinkles (visible by AFM only, marked with red arrows) serving as the source of the parallel wrinkle formation.

Major and minor wrinkles (2D FFT filtration) and wrinkle propagation geometry (2D FFT)

The long-range (major) and short-range (minor) wrinkles can be separated well using 2D FFT filtration. An example of this procedure is shown in Fig. S5, where the topography image of the GN@pillars_A (Fig. 3a) is split into long-range (Fig. 3b) and short-range (Fig. 3c) wrinkles.

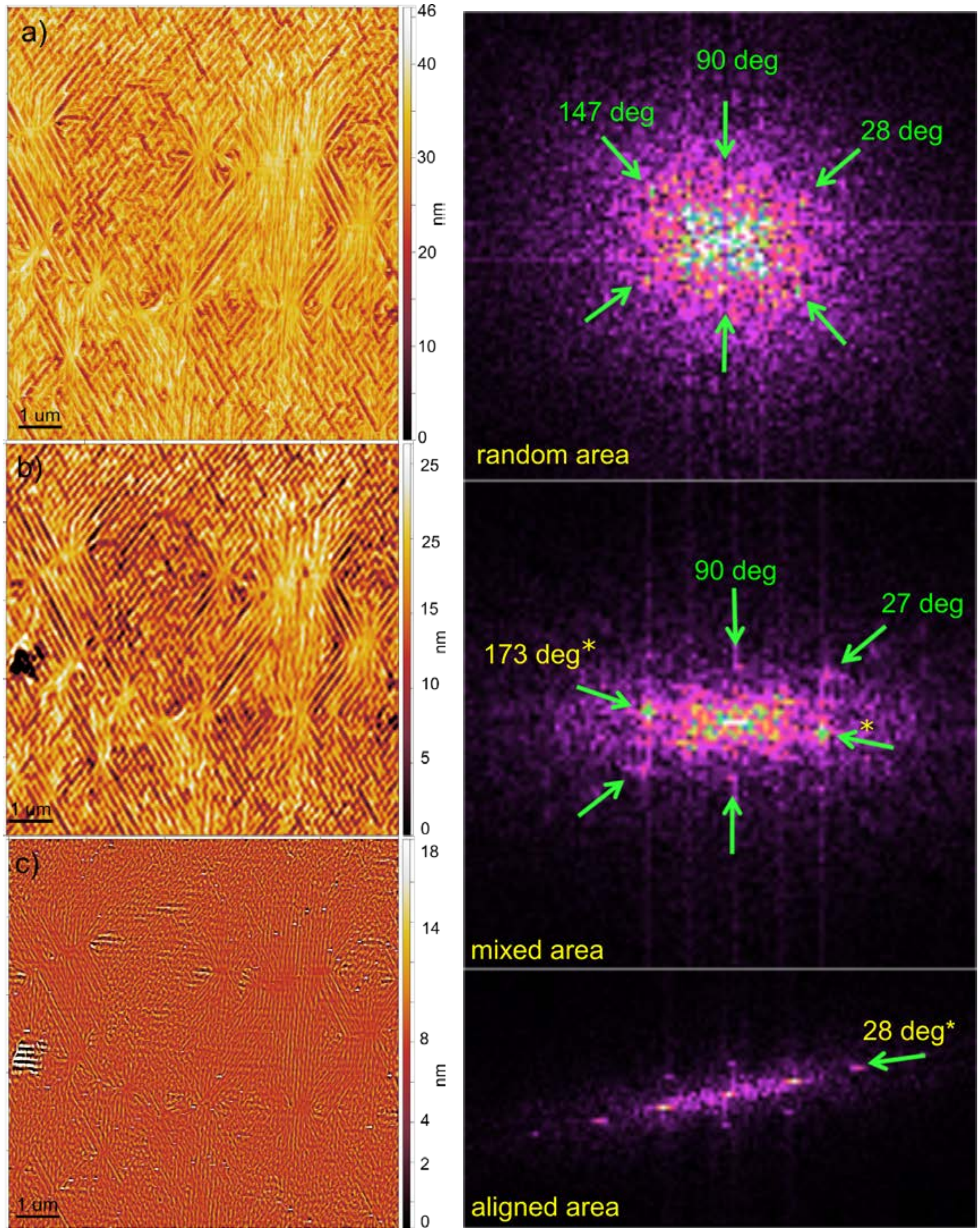

Figure S5. Left column: the AFM topography image (a) of the GN@pillars_A sample with the long-range (b) and short-range (c) frequency components separated using the 2D-FFT filtration. Right column: Example of the 2D FFT images for typical wrinkle areas, demonstrating symmetry of wrinkle propagation and visualising the angles between main propagation directions. Yellow star marks directions with highest intensity, hence the most preferred direction of wrinkle propagation for individual wrinkle area type.

Position of wrinkles with respect to the nanopillars and layer corrugations

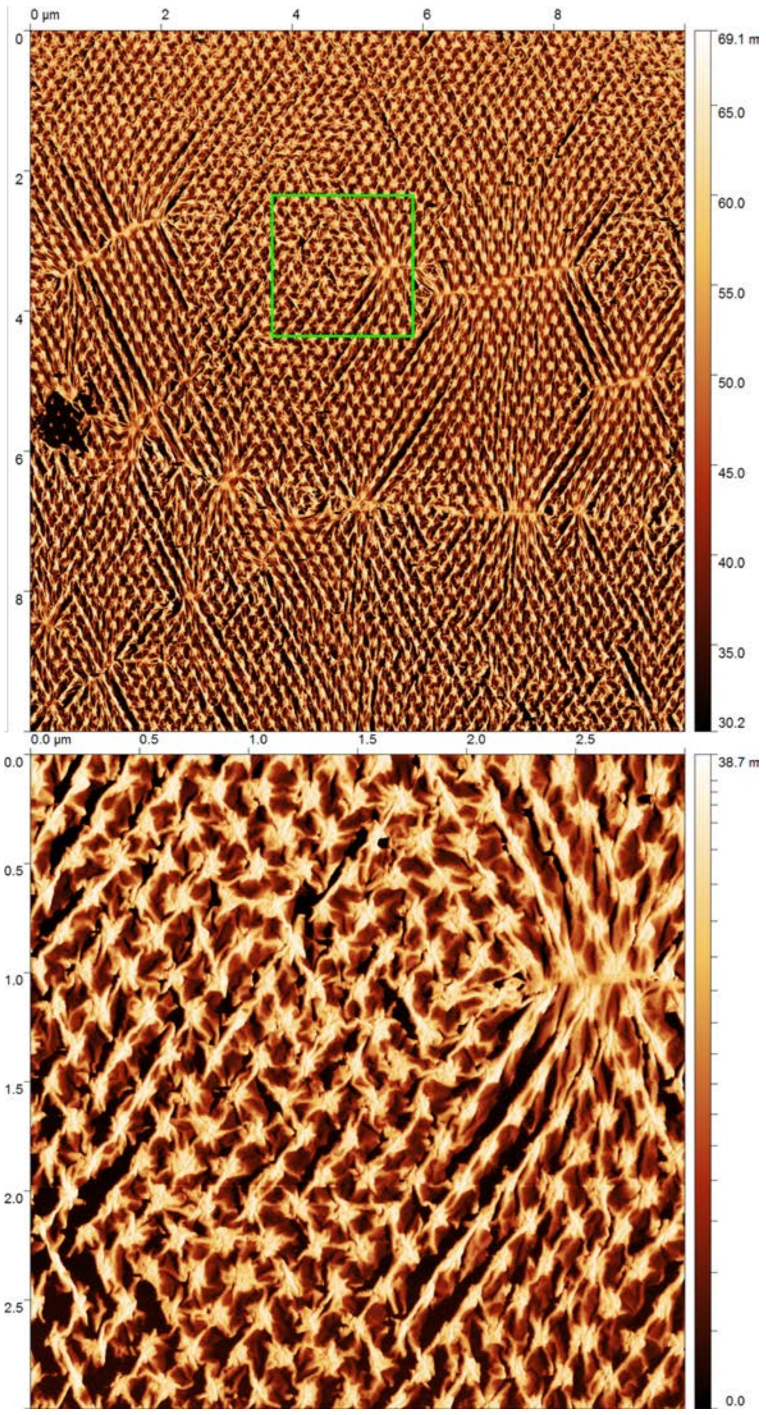

Figure S6. Visualisation of the wrinkle location with respect the nanopillar positions for image containing all three specific wrinkle areas. Image was created as a subtraction of the AFM topography and phase images - AFM topography image shows the complete wrinkle network, whereas the phase image visualises both the topography corrugations (wrinkle peaks, graphene line boundaries) as well as the objects hidden beneath the graphene layer which are in direct mechanical contact with the graphene (pillar tops). Combination of both images via image arithmetic allows visualisation of topography together with mechanical perturbations of scanned surface.

Line boundaries – evidence for graphene on flat substrate

The area of the graphene transferred on the substrate is approximately 1cm^2, whereas the central part of the sample where the nanopillar array is located is only 1mm^2 large. Graphene transferred on the bare Si/SiO_2 substrate is used as an area to explore origin of the thin lines on the phase images.

Figures S7, 8 and 9 serve as a direct evidence that lines with different phase contrast than the rest of the phase image (mostly negative for the figures in the manuscript body) are attributed either to wrinkles observable on the topography, or to graphene grain boundaries. The line defect visible on the phase image, which is continuous and forms structures connected with other phase line defects, is the grain boundary. There are no other possible sources that are visible in the phase image and not in the topography.

To see which line defects on phase are also manifested on the topography images, we compared masked wrinkles and masked phase images. Applying the image arithmetic, overlapping regions of topography and phase corrugations are well observable (Figures S7e, S8c, f, i).

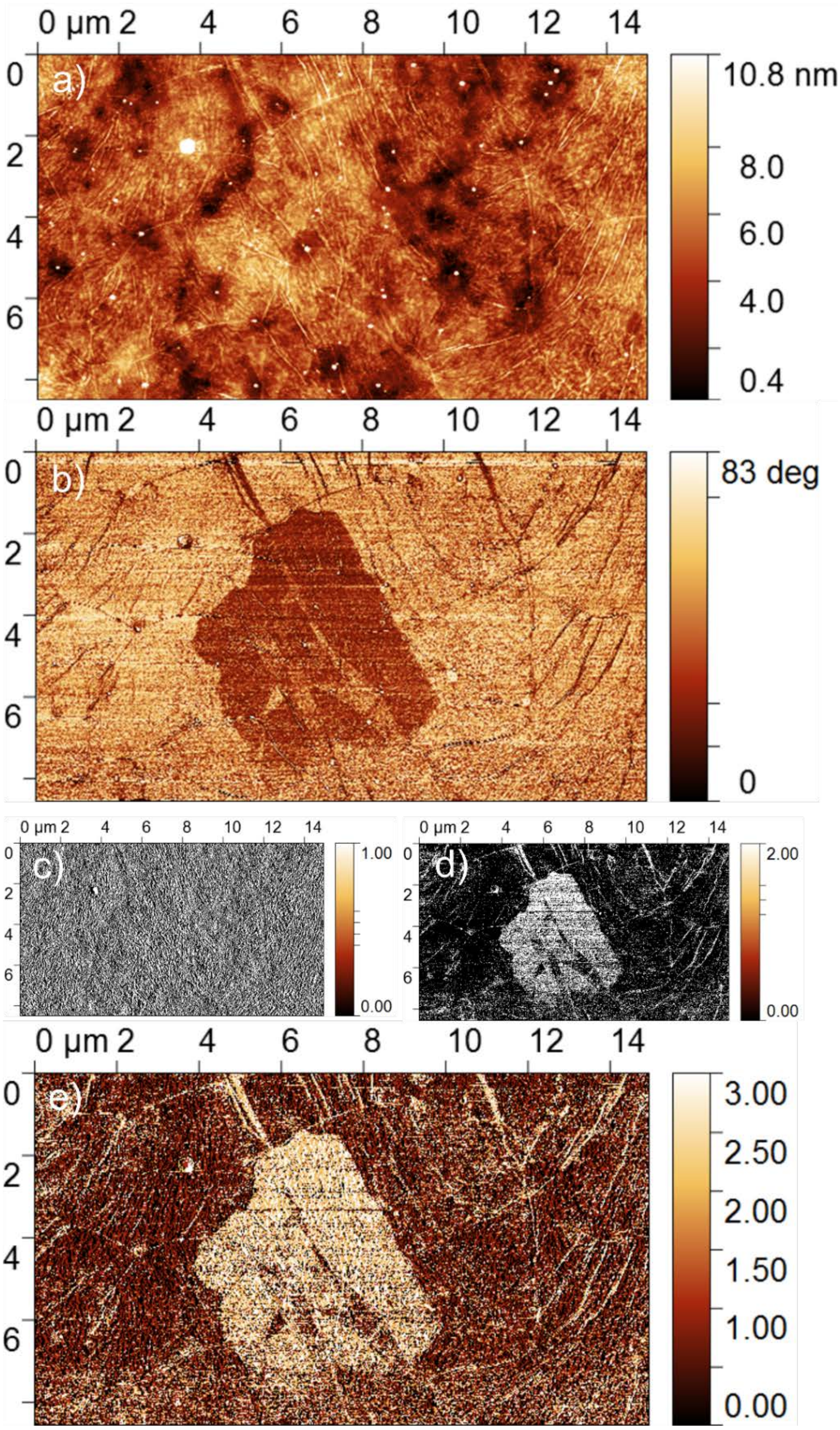

Figure S7. Example of the edge of graphene sample, showing wrinkled structure of graphene on bare Si/SiO_2 substrate. Wrinkles do not form perfectly ordered networks, as was observed for pre-patterned nanopillar arrays. a) AFM topography, b) phase image with an individual graphene grain in the central part of image, c) extracted masked wrinkles (white), d) extracted mask of phase shift, e) sum of c and d). Intersecting regions (lines) observable both on topography and phase are in white and correspond to the wrinkles visible on the phase image. Orange areas belong only to phase contrast and do not correlate with any topographical features (so we still see the GN grain, but covered with wrinkles in white), red is attributed to wrinkles.

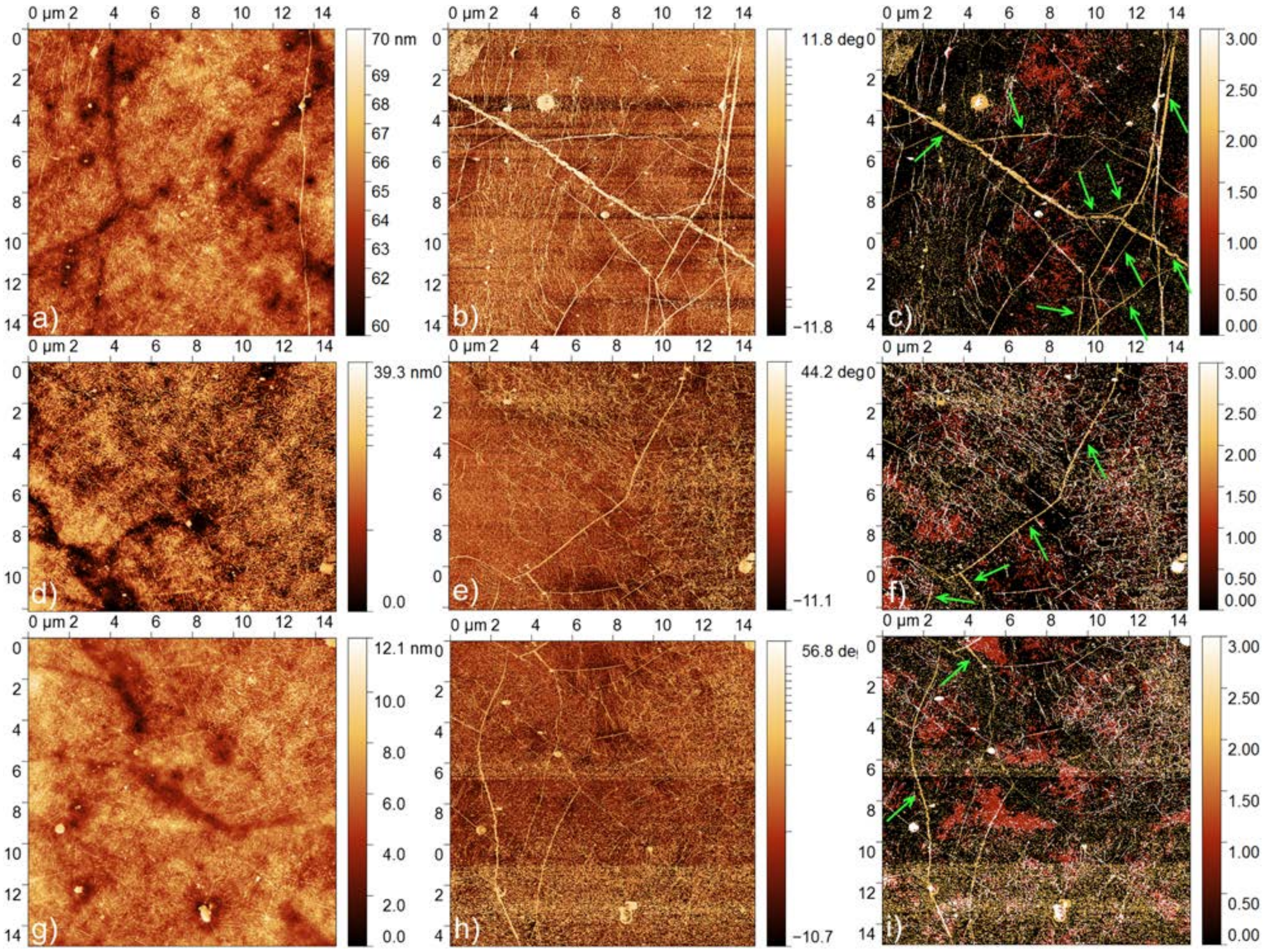

Figure S8. Example of three different regions of wrinkled graphene on Si/SiO_2 substrate. Left (a, d, g): AFM topography images, centre (b, e, h): phase image, right (c, f, i): sum of the masked wrinkles and features on phase images, which correlates the topography with the phase. It is clearly visible that many lines in the phase image are connected to features in the topography (white colour in the c, f, i images). They can thus be attributed to the thin (transferred) wrinkles. Those lines manifested only on the phase images (marked by green arrows), and are not visible on the topography have orange contrast in the sum image (c, f,i) and can be attributed to the graphene grain boundaries. Besides the grain boundary, there is no other source which can create a line defect in GN, and is only visible in the phase contrast map.

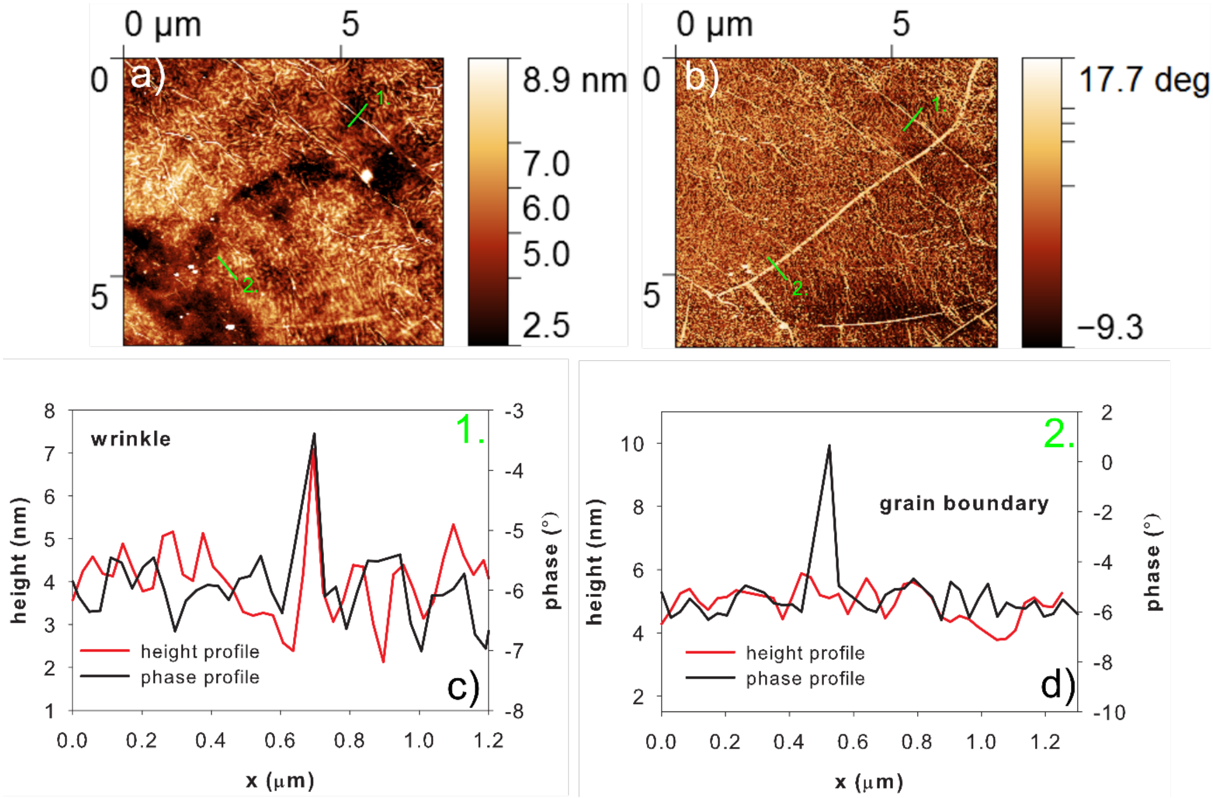

Figure S9. Example of the profile of wrinkle (c) and grain boundary (d) on the AFM topography (a) and phase (b) images.

Mechanical properties mapping

We mapped samples using the PF-QNM method, where simultaneously the AFM topography, adhesion and deformation can be measured. Deformation was correlated with the topography; it can be seen in Figures S10 – S13 that deformation of the regions with free standing (suspended) graphene is much lower than the deformation of supported graphene on nanopillars. Moreover, deformation reached for graphene in the GN@pillars_A sample is much larger than deformation reached in the GN@pillars_B sample, pointing to higher strain of the graphene layer and better adhesion of graphene to larger pillars. Larger strain of the layer should lead to smoothening of wrinkles and more flat-like profile of graphene layer, which was observed for the GN@pillars_B sample. Better attachment of the graphene to pillars for the GN@pillars_B sample was indirectly confirmed with adhesion, which is higher than for the GN@pillars_A sample (Figures S12 and S13).

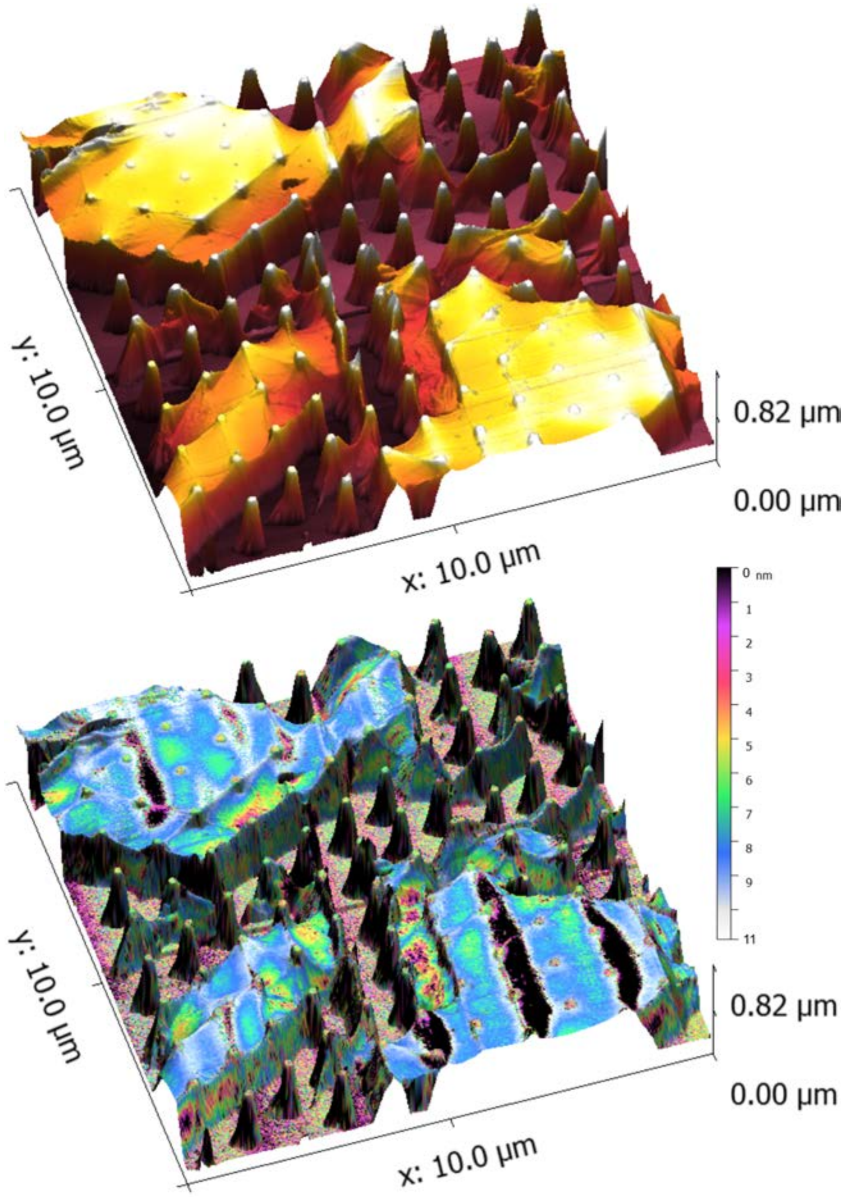

Figure S10. 3D AFM topography (upper image) and deformation (lower image) map of the GN@pillars_B sample.

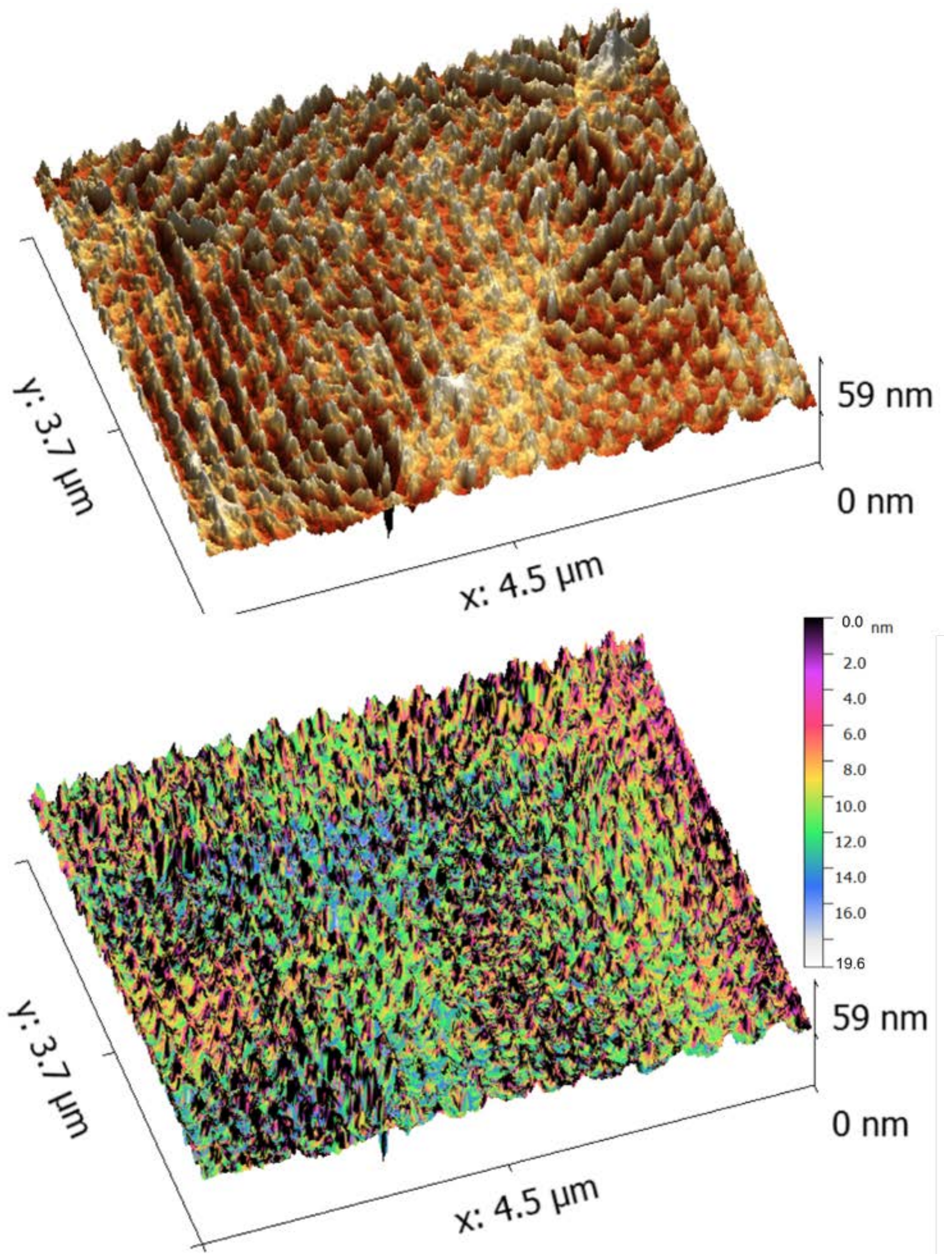

Figure S11. 3D topography (upper image) and deformation (lower image) map of the GN@pillars_A sample.

Mechanical properties mapping - role of adhesion and deformation in formation of phase contrast

Figure S12 and S13 show that phase contrast for the wrinkles located in between pillars is dominated by adhesion, whereas pillar regions are dominated by deformation.

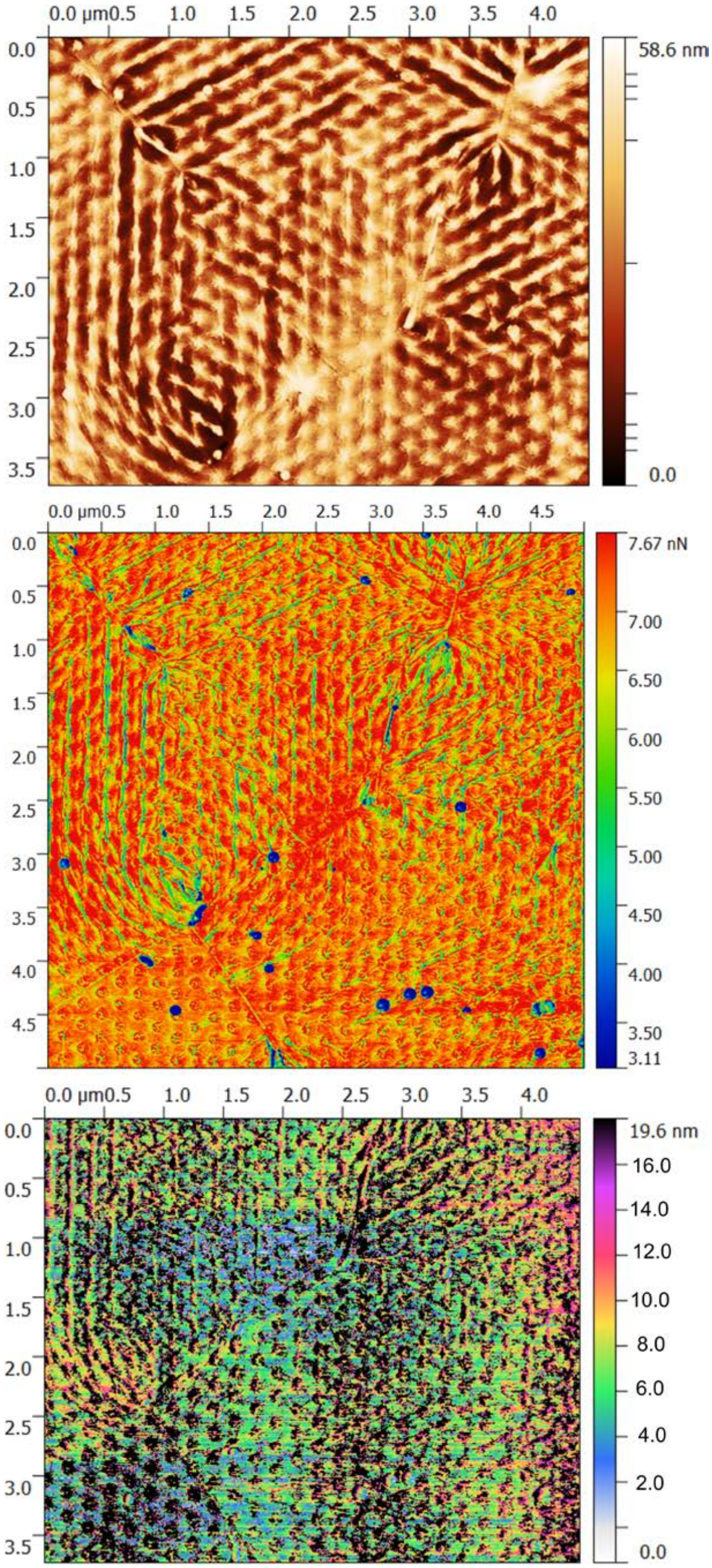

Figure S12. a) Topography, b) adhesion and c) deformation for the GN@nanopilars_A sample.

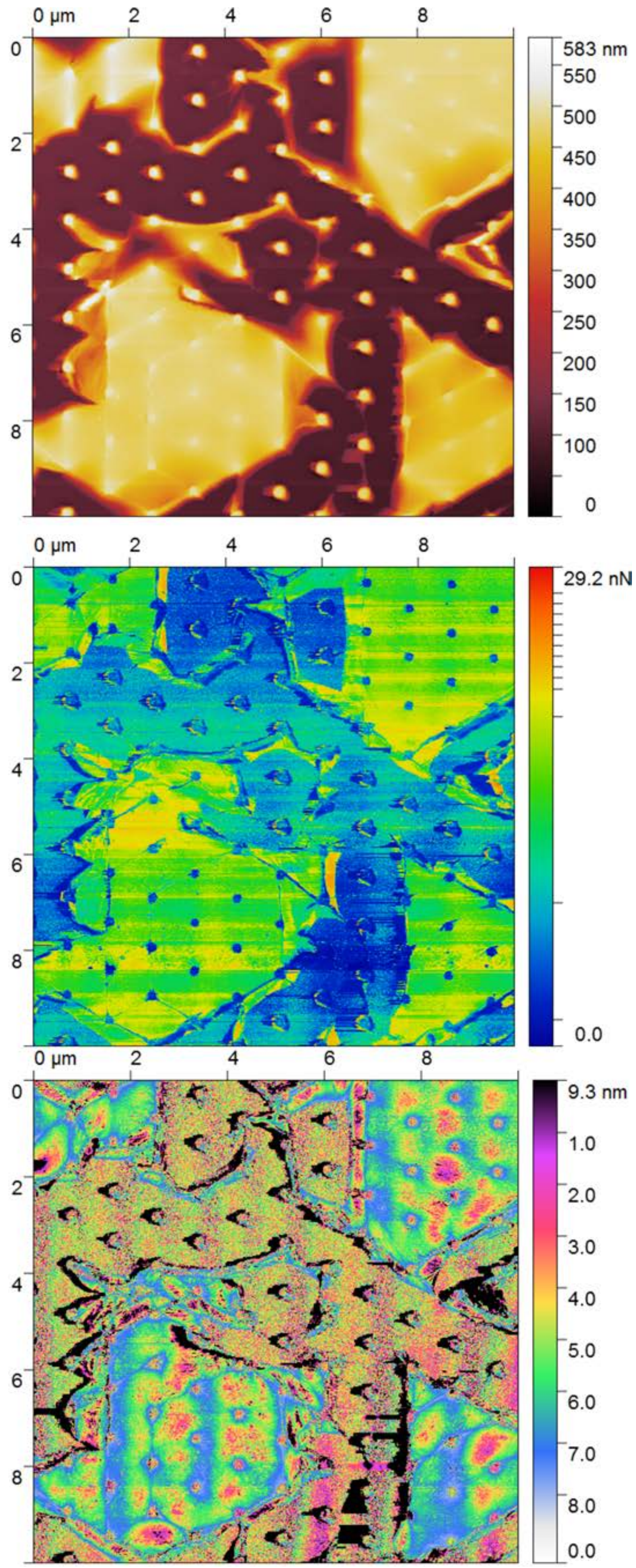

Figure S13. a) Topography, b) adhesion and c) deformation for the GN@nanopilars_A sample.

Calculation of the peak-to-peak amplitude of the layer (R_{tm})

To calculate the median peak-to-peak amplitude of the graphene layer R_{tm} (median distance between wrinkle height and inter-wrinkle valley depth) for individual area types, we built procedure for the MATLAB network.

Calculation of the R_{tm} parameter (Eq. 1 in the manuscript file) works as follows. The individual area types (random, aligned and mixed wrinkle areas) were cut out from the large topography AFM images, producing images with *m* x *n* pixels. Then all the peak maxima (p_i) and minima (v_i) were found for each individual line *m* in the image. Finally, the median R_{tm} was calculated from median peak height (p_m) and valley depths (v_m) of whole area using Eq. 1. Example of the one image line of the 'mixed area' with highlighted p_i and v_i is depicted in Figure S14.

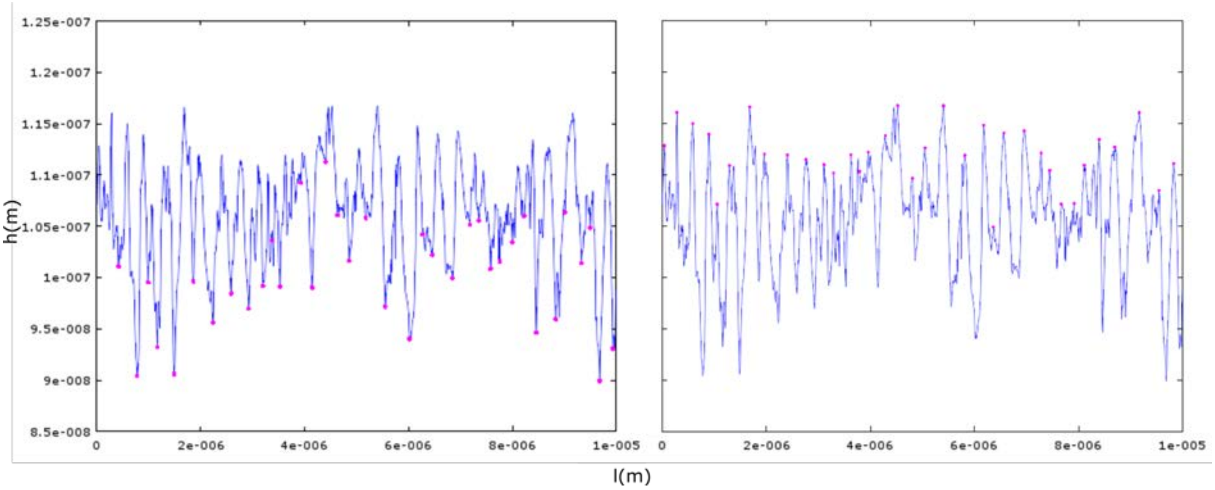

Figure S14. Example of line cuts for the 'mixed area' with marked valleys (left) and peaks (right). Purple spots represents found peak maxima (p_i) and minima (v_i).

Large scale images of the prepared nanopillar arrays

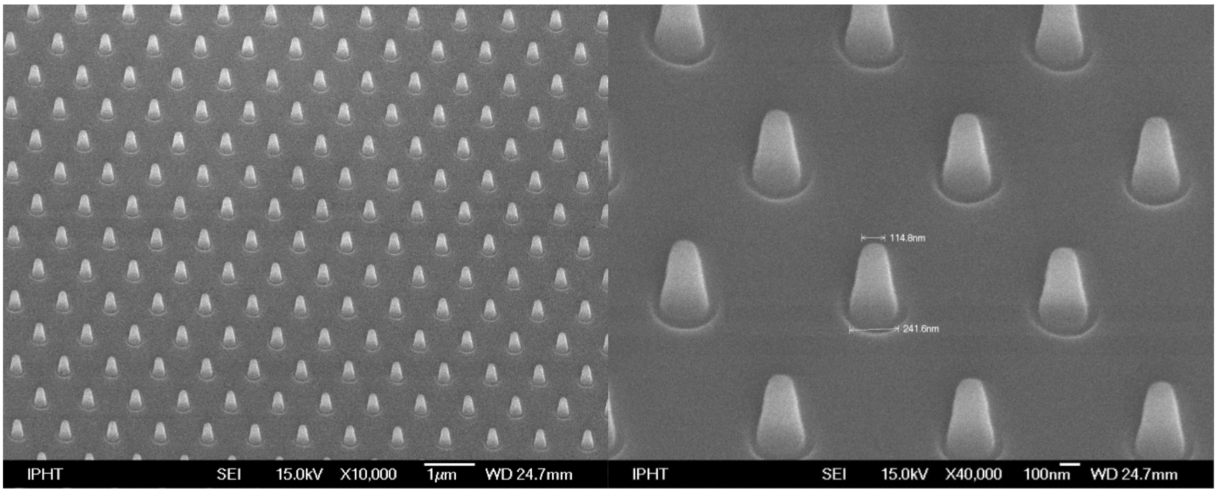

Figure S15: 25° tilted SEM images of the hexagonal nanopillar gratings with a pitch of 1000 nm. The diameter of the SiO_2-nanopillars is about 110 nm (on top), and their total height is about 460 nm (~100 nm over-etched into the Si-wafer).

Additional comments on the Raman spectroscopy data and analysis

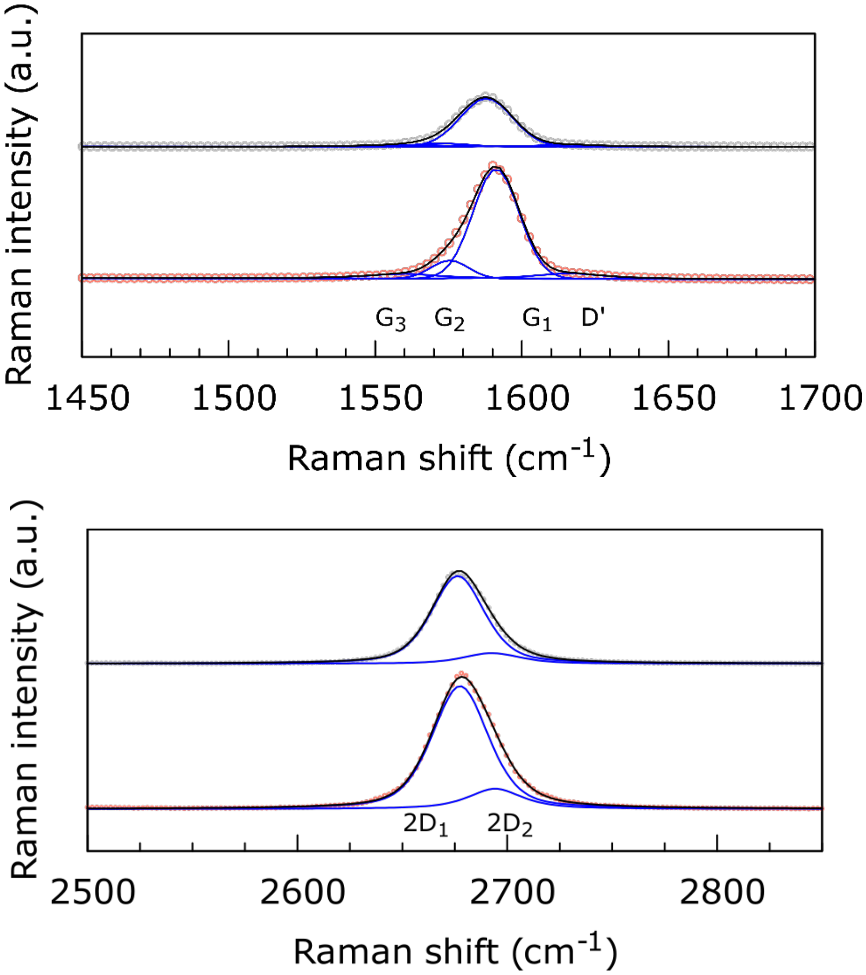

Figure S16: Close up of the G mode (top panel) and 2D mode (lower panel) regions, magnified from Fig. 2 in the main text, of typical Raman spectra of the GN@pillars_A sample for regions with randomly wrinkled graphene (grey data points) and aligned wrinkles (red data points). It is clearly visible that the G mode of the random area (grey data points) is rather symmetric, whereas the aligned area (red data points) clearly exhibits an asymmetric G mode. This asymmetry is fitted using a larger G_2 component and one, although small, extra peak function (G_3) that accounts for the long wrinkles.

Figure 16 shows the close-ups of the G and 2D regions (from Fig. 2 in the main text) of the GN@pillars_A sample. As indicated in the main text, the strain differences were estimated through the analysis of the 2D' mode. However, several interesting observations can be made regarding the shape of the G mode, especially concerning its asymmetry in the spectra from the aligned wrinkle areas. The larger asymmetry usually indicates the presence of regions with more defined state (doping/strain) of the graphene within the laser spot [S1]. If the regions are not so well defined, in other words there are many areas with only slightly differing strain/doping within the spot, then the G peak becomes simply broader and shifted towards the average values. We can see this is indeed the case of the difference between the aligned and random areas in our GN@pillars_A sample. The doping and strain (level and

direction) are much more random in the area where the wrinkles are oriented randomly, while there is a limited number of strain/doping configurations with specific Raman shifts in the aligned area. On the other hand it is difficult to draw any further conclusions, for example from the widths of the particular components, since apart from homogeneity of the sample, both the doping and strain influence the width of the G mode.

[S1] Vejpravova, J. *et al*. Graphene wrinkling induced by monodisperse nanoparticles: facile control and quantification. *Sci. Rep.* **5,** 15061 (2015).
